# Quantum Mechanics MP2 and CASSCF Study of Coordinate Quasi-Double Bonds in Cobalt(II) Complexes as Single Molecule Magnets

**DOI:** 10.3390/nano15120938

**Published:** 2025-06-17

**Authors:** Yuemin Liu, Salah S. Massoud, Oleg N. Starovoytov, Tariq Altalhi, Yunxiang Gao, Boris I. Yakobson

**Affiliations:** 1 Department of Chemistry, Prairie View A&M University, Prairie View, TX 77446, USA; yugao@pvamu.edu; 2 Department of Materials Science and NanoEngineering, Rice University, Houston, TX 77005, USA; 3 Department of Chemistry, University of Louisiana at Lafayette, Lafayette, LA 70504, USA; salah.massoud@louisiana.edu; 4 Department of Chemistry, Faculty of Science, Alexandria University, Moharam Bey, Alexandria 21511, Egypt; 5 Center for Computation and Technology Services, Louisiana State University, Baton Rouge, LA 70803, USA; olegs@lsu.edu; 6 Chemistry Department, Taif University, Taif 21974, Saudi Arabia; ta.altalhi@tu.edu.sa

**Keywords:** coordinate quasi-double bond, cobalt (II) complex, tripod ligands, single molecule magnet, second order perturbation theory analysis, zero field splitting (ZFS), magnetic susceptibility, dispersion force

## Abstract

Co(II) complexes have shown promising applications as single-molecule magnets (SMMs) in quantum computing and structural biology. Deciphering the Co(II) complexes may facilitate the development of SMM materials. Structural optimizations and calculations of chemical and magnetic properties were performed for Co(II) complexes with a tripodal tetradentate phenolate-amine ligand using MP2/aug-cc-pvdz, MP2/Def2svp, and CASSCF/Def2svp methods. The Second Order Perturbation Theory Analysis of Fock Matrix in NBO Basis unravels that Co(II) ions form unusual coordinate quasi-double bonds with ligand oxygen donor atoms, and the bond strengths range from 142.01 kcal/mol to 167.36 kcal/mol but lack further spectrometric evidence. The average 151.70 kcal/mol of the Co(II-O coordinates quasi-double bonds are formed mainly by two lone pairs of electrons from the ligand phenolate donor oxygen atoms. Dispersion forces contribute 24%, 28%, 27%, and 31% to the Co(II)-ligand interaction. Theoretical results of ZFS D, transversal ZFS E, and g-factor agree well with the experimental values. Magnetic susceptibility parameters calculated based on 5 doublet roots account for 85% of results computed 40 doublet roots are specified. These insights may aid in the rational design of SMM materials and Co(II) porphyrin fullerene conjugate for CO_2_ electroreduction with superior magnetic properties.

## 1. Introduction

Single-molecule magnets (SMMs) refer to the category of lanthanide complexes [1,2,3,4,5,6] and transition metal complexes [5,6,7,8,9,10,11] exhibiting slow magnetic relaxation and magnetic hysteresis [10,12]. With these attributes stemming from a pure molecular origin, SMMs show fundamental differences from regular bulky magnets [13]. Some transition metal ions are classified as single ion magnets (SIMs) because of magnetization resulting from 3d orbitals [14]. The SMMs have been pursued in the applications in information storage [15], quantum computing [16], spintronics [17], and structural biology [18,19,20]. Since retaining magnetization after exposure to an applied permanent magnetic field serves as the molecular basis for the applications of SMMs [21], high effective energy barriers (U_eff_) for spin reversal of molecular magnetic moments are required for the paramagnetic complexes which originated from high magnetic anisotropy (D). The D tensor accounts for the axial component of the magnetic dipole–dipole interaction in Zero Field Splitting (ZFS) after degeneracy removal in the absence of a magnetic field. The SMMs with spin transitions at gigahertz (GHz) level at low magnetic fields are also called Kramer complexes. The magnetic anisotropy barrier can be defined by U_eff_ = |D|S^2^ (D refers to ZFS, and S is the total electron spin quantum numbers) for non-Kramer SMM molecules with an overall integer spin larger than ½, whereas the energy barrier can be given with Ueff = |D|(S^2^ − 1/4) for Kramer SMM complexes with half-integer spin [22,23]. Recently, it was found that the D indices do not have to be negative for slow magnetization relaxation in SMMs, as there are cases of SMMs with both positive and strong easy-plane anisotropy [6,24], which is true for the Co(II) complexes [25,26,27,28,29,30,31,32,33,34,35,36]. The common spin reversal demagnetization relaxation nanosecond scale of SMMs is mainly controlled by spin-lattice, magnetic dipole, and Zeeman, as well as spin–spin interaction [37]. The major spin-lattice relaxation reverses magnetization by crossing energy barriers that accompany the thermal energy exchange between the spin and lattice. If the magnetic anisotropy barrier energy U_eff_ is significantly larger than the thermal energy kT, then the magnetization can be maintained for a relatively long time. The spin-lattice relaxation is normally driven by three phonon-involved mechanisms, which are the Direct, Orbach, and Raman processes [37], among which the Orbach mechanism plays a predominant role in the spin-lattice relaxation [32]. The Orbach spin-lattice mechanism occurs with a relaxation rate of U_eff_ Arrhenius dependence [23,38]. The dynamics of the SMMs vary strongly with the temperature. The magnetic moments of SMMs usually fluctuate quickly, whereas magnetic variation is almost blocked at low temperatures. The blocking temperature refers to the temperature at which the relaxation time (τ) of the systems is 100 s for SMMs. The effective spin-reversal barrier and blocking temperature serve as two major criteria for examining the performance of SMMs [19,39].

**Figure 1:**
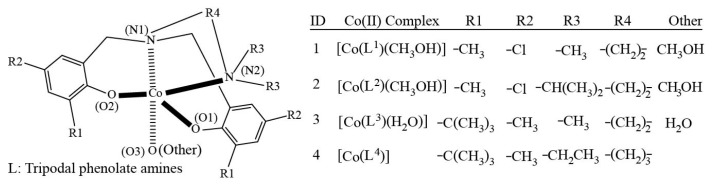
Structures of four Co(II) complexes based on the ligand tripodal tetradentate phenolate amine ligands.

The rational design of SMMs has been focused on enhancing the U_eff_ in static crystal field through polynuclear complexes, which consist of mononuclear fragments with high spin [40]. Since spin larger than 12 would not increase the magnetic anisotropy energy barrier anymore [41], the alternative strategy lies in boosting magnetic anisotropy by adjusting ligand geometry and symmetry [42]. A ligand environment can inhibit quantum tunneling of magnetization (QTM), which in turn gives rise to high magnetic degeneracy D [43,44,45,46]. The bulk groups were reported to induce spin-crossover (SCO) of Co(II) complexes by combining with environmental factors, including light, temperature, pressure, and other molecules [47]. Geometric distortions of complexes with low symmetry may cause effects on orbital degeneracy and orbital angular momentum contribution in the total magnetic moment to achieve a large magnetic anisotropy [48,49,50]. Cobalt(II) ion is capable of forming a broad variety of complexes, including 2-coordinate linear complexes [51,52], 3-coordinate trigonal planar complexes [53], 4-coordinate tetrahedral or pseudo-tetrahedral complexes [8,54,55,56,57,58], 5-coordinate distorted square pyramidal [23,59] or trigonal bipyramidal [50,60,61,62], 6-coordinate octohedral complexes [63,64,65,66,67,68,69], 7-coordinate pentagonal bipyramidal and capped trigonal prism [70,71,72], 8-coordinate square-antiprismatic [73,74,75]. Extensive efforts led to the discovery of Co(II) complexes with U_eff_ around 300 cm^−1^ [60,76], with the highest U_eff_ of 450 cm^−1^ in the absence of an applied magnetic field [51]. The pseudo-tetrahedral arrangement enables strong spin–orbit coupling between the ground and excited electronic states. Therefore, Co(II)-based SMMs with N_2_O_2_ coordination donor sets were demonstrated to show slow magnetic relaxation at zero or DC magnetic fields. The relative orientations of d-orbitals for four coordinate Co(II) complexes can vary with the distorting of coordinating arrangments [56,57,58]. Four Co(II) complexes designed are described in Figure 1, with an N_2_O_2_ coordination tripodal phenolate amine donor and distorted pseudo-tetrahedral structure, have been synthesized and revealed SMM behaviors [60,77]. A Co(II) porphyrin fullerene conjugate plays a catalytic role in the CO_2_ electroreduction [78,79]. Quantum mechanical (QM) simulations enable an understanding of intramolecular interactions of transitional metal complexes [9,80,81], so QM calculations will be pursued to investigate the effects of ligand molecule adjustments on magnetic properties of Co(II) complexes. Insights into Co(II) complex electronic structures specifcally coordinate bond enable the further development of SMMs materials and Co(II) porphyrin fullerene hybrid systems for CO_2_ electroreduction.

## 2. Materials and Methods

Structural models of four cobalt complexes were built based on the crystallographic structure [77], which are depicted in Figure 1. Computational simulations of the four compounds were performed using Gaussian-16 [82] and the ORCA software package (version 5.01) [83]. Structural optimizations of the four complexes were carried out using Becke–Perdew (BP86) functional [84,85,86] and Karlsruhe basis sets of the valence double-ζ basis set with polarization functions (def2SVP) on main-group and transition-metal elements [87]. The resolution of identity and chain of the sphere (RIJCOSX) approximations [88] were employed in conjunction with a variety of auxiliary basis sets to fit Coulomb’s potential [89]. An auxiliary basis set -/JK type was applied for structural optimization.

After structural optimization, the unrestricted open-shell MP2/Def2SVP calculations were conducted for the computations of intramolecular interaction and natural bond analysis of the complexes, followed by evaluations of the spin contaminations using the Gaussian-16 program.

The optimized geometries were also used for multiconfigurational (CASSCF) calculations of the Spin–Hamiltonian (SH) parameters and the magnetic properties of the complexes [90]. The minimal activity was applied for seven electrons 3d^7^ of Co(II) ion. The roots of states stemming from the quartet and doublet multiplicities were considered for the 3d^7^ configuration. There were 10 quartet roots for 4F and 4P and 40 doublet roots, which are 2G, 2P, 2H, 2D, 2D, and 2F. These roots were taken into consideration with the same weights, and then the CASSCF computation was implemented as state-averaged CASSCF. The dynamic correlation effect was considered using the highly contracted N-electrons valence perturbation theory to second order (NEVPT-SC) [91]. The DKH formalism was applied to account for the scalar relativistic effect in the structural optimization and calculations of magnetic properties. The mean-field approximation was made to take spin–orbit coupling into account.

The spin Hamiltonian (SH) parameters were extracted for 1–4, fitting both the *χ_M_T* vs. *T* and M(H) data using PHI software version 3 [92]. To prevent any overparameterization, only an isotropic g factor and spin Hamiltonian are considered in the fittings, with some corrections for the intermolecular interactions at low temperatures for the complexes.

## 3. Results and Discussions

### 3.1. Structural Parameter and Molecular Geometry

**Figure 2:**
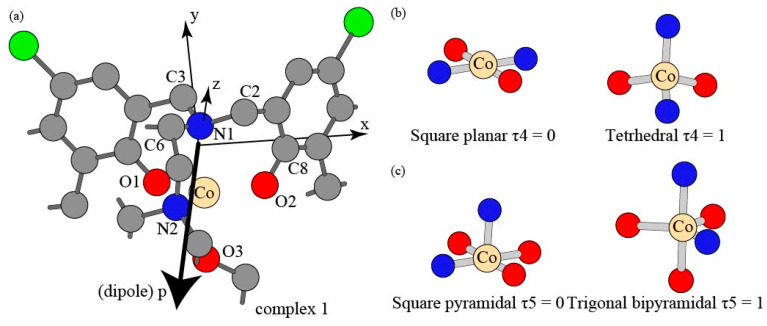
Structure of complex 1 with hydrogen atoms omitted for clarity and d orbital diagrams of the geometries for 4-coordinate and 5-coordinate complexes. (**a**) Structure of complex 1 with hydrogen atoms omitted for clarity. (**b**) The geometries for 4-coordinate complexes. (**c**) The geometries for 5-coordinate complexes.

**Figure 3:**
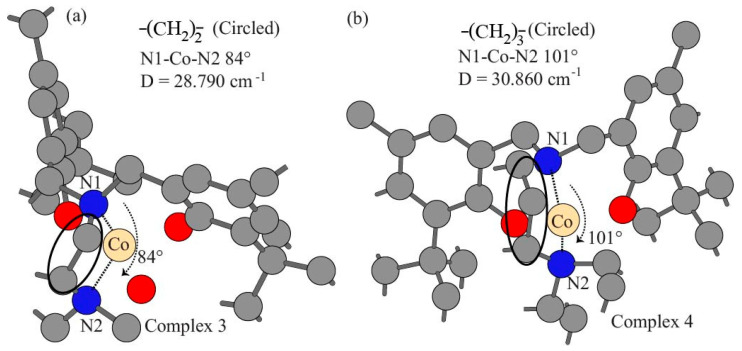
Comparison of the N-Co(II)-N angles between complexes 3 and 4. (**a**) The N-Co(II)-N angles in complex 3. (**b**) The N-Co(II)-N angles in complex 4.

The spin operator values in the single point MP2/Def2SVP calculations of the four complexes are included in Table 1. The cobalt-metal-ions-to-ligand (M-L) donor bond lengths, cobalt-ions-to-ligand (L-M-L) donor bond angles, geometric indices τ of the four complexes, natural bond orbital (NBO) charges for ligand donor atoms and cobalt(II) ions are listed in Table 2, Table 3, Table 4 and Table 5, respectively. The optimized geometry for complex 1 is depicted with the hydrogen atoms hidden for clarity in Figure 2a, and the two extremes for the 4-coordinate pseudo-tetrahedral chromophore and the 5-coordinate distorted trigonal bipyramidal structures are illustrated in Figure 2b and Figure 2c, respectively. The structural effect of the bridge length (CH_2_)_2_ and (CH_2_)_3_ between two nitrogen donors on the 5-coordinate complex 3 and 4-coordinate complex 4 is shown in Figure 3. Unrestricted open shell MP2 calculations allow α spin and opposite β spin electrons to have two sets of orbitals, respectively. Spin contamination occurs due to the fact that the wavefunction calculated from the unrestricted procedure may deviate from the eigenfunction of the overall spin operator <S^2^>. Spin-contaminated wavefunction can cause errors in energies, geometries, and population analyses [93]. The Gaussian-16 contains an annihilation process to lower the spin contamination. Our calculations produce a spin operator S^2^ value of 3.7575 before annihilation and 3.75 after annihilation, which are very close to the actual 3.75 manually calculated by the equation S^2^ = s(s + 1) = 3/2(3/2 + 1) = 3.75 where s represent the spin of system. Therefore, the simulation results of our work demonstrate low spin contaminations and high accuracies. Table 2 indicates that the N1-Co(II) bond lengths range from 2.06 Å to 2.20 Å, and the O-Co(II) bond length ranges can be largely categorized as those with tripodal phenolate amine (1.90 Å to 1.97 Å) and those with small molecule methanol or water (2.11 Å to 2.13 Å). While complexes 1 to 3 show relatively close N1-Co(II) bond parameters of 2.19, 2.17, and 2.16 Å, respectively, complex 4 has the shortest N1-Co(II) bond length of 2.06 Å. The N2-Co(II) bond length parameters share a similar range from 2.06 Å to 2.20 Å to that for N1-Co(II) of the complex series with an exception for the two slightly short 2.12 Å and 2.11 Å for ligand 1 and ligand 3, respectively. The Co(II)-N bond lengths of the four complexes fall in the ranges of other experimental results [94]. The bond lengths of O-Co(II) (1.90 Å to 1.97 Å) involved with the ligand are shorter than both N-Co(II) and O-Co(II) with small molecules of methanol or water. These values are also shorter than the Co(II)-O coordinate bonds of 1.952 to 2.239 Å were reported by Kim et al. [95]. The Co(III)-O bond length of 1.90 Å was observed in the pressure-response study [96]. Therefore, the Co(II) ion probably forms relatively stronger with ligands than with methanol, and complex 4 has the most compact structure for its four shortest without a fifth coordination bond with methanol in the four complexes. Table 3 shows that there are six coordination bond angles for 4-coordinate complex 4, and ten coordinate bond angles exist for 5-coordinate complexes 1 to 3. The most obvious difference lies in the bond angle N1-Co(II)-N2 for the four complexes. For the first 1–3 complexes, the short linker -(CH_2_)_2_- between the two ligand donor nitrogen atoms leads to the bond angle values of N1-Co(II)-N2 of 82°, 83°, and 84° for complexes 1−3, respectively. On the other hand, complex 4 shows an N1-Co(II)-N2 angle of 101° due to a long bridge -(CH_2_)_3_- between the two donor nitrogen atoms, as indicated in Figure 1. The bond angles are usually expressed in geometric parameters or indices, which are more informative [97,98,99]. Just like the three octahedral distortion parameters for describing the twisted octahedral geometry [97,100,101,102] caused by the Jahn–Teller electron–photon coupling for reducing symmetry [103], the two largest coordination bond angles can be used to derive geometric index τ values for evaluating the structural deviation from tetrahedral and trigonal bipyramidal for complexes 1 to 4. The τ_4_ values of 4-coordinate complexes range from 0 for square planar geometry to 1 for tetrahedral structure (τ_4_ = −0.00709α − 0.00709β + 2.55, α and β represent the two largest angles out of six bond angles) [99,104]. The τ_5_ values range from 0 for the square pyramidal structure to trigonal bipyramidal geometry (τ_5_ = −0.01667α + 0.01667β, α and β are the two largest angles out of ten bond angles) [98]. These parameters include τ_4_ of 4-coordinate for Co(II) 1 to 4 while the small ligands methanol or water are ignored in complexes 1 to 3 and τ_5_ for 5-coordinate values for complexes 1 to 3. Table 4 shows that τ_4_ increases from 0.75623, 0.76332, 0.79877, and 0.82004 for complexes 1 to 4, respectively. Complexes 1–3 have distorted tetrahedral structures when coordinating bonds with methanol or water are neglected, and complex 4 exhibits the closest geometries to a tetrahedron. Considering the Co(II)-O with methanol or water renders the distorted trigonal bipyramidal structures with the geometric factors τ_5_ of 0.75015, 0.76682, and 0.73348 for compound 1 to 3, respectively. It is known that the complexes with coordination 4 can adopt geometries from square planar to tetrahedral geometries, which are illustrated in Figure 2b, and 5-coordination complexes may have geometries from square pyramidal to trigonal bipyramidal geometries, which are illustrated in Figure 2c. The d-orbital energy splitting of tetrahedral complexes is relatively small even if the coordinated ligand molecules exhibit strong crystal field because of lower coordination number. The d-orbital splitting constant usually does not exceed spin pairing energy, so electrons occupy the high energy d-orbitals instead of pairing up in the low energy d-orbitals. As a redult, tetrahedral complexes exhibit high spin configurations. With a same coordination number of 4, the sqaure planar strcutures have significantly lower energies of dxz, dyz, and dz^2^ due to the lack of electron repulsion along *z*-axis. In the square plane configuration, the dx^2^-y^2^ has the highest energy and the dxy orbital shows a slightly lower level. The square planar complexes always have large crystal field splitting for low spin configuration because of equatorial coordination bond. The deviation from tetrhedral to square planar geometry most likely leads to a relatively stronger crystal field splitting. According to hole (empty orbital) formalism, the tetrahedral goemetry with d^n^ electrons shares a same symmetry with a oectahedral configuration of n holes (empty orbitals) for the splitting of the d-orbitals for tetrhedral complexs occurs oppositely to that for the octahedral geometry [105]. The NBO charges are assigned based on a localized representation of electron density, which enables a more accurate description of atomic partial charges [106,107]. Table 5 exhibits a largely normal pattern of NBO partial charges of N1, N2, O1, and O2 for the four Co(II) complexes, with a little exception that −0.40 a.u. of O1 and −0.41 O2 for complex 1 are 0.08 a.u. and 0.07 a.u. lower than 0.48 a.u. for all other coordinating oxygen atoms. The NBO partial charges for coordinating nitrogen range from 0.32 a.u. to 0.35 a.u., consistently lower than those for oxygen atoms. Overall, Co(II)-O (ligand) bond lengths are relatively shorter than the corresponding Co(II)-N bond distances. Co(II)-O (MeOH) in complexes 1–3 and Co(II)-O and Co(II)-N bonds in complex 4 represent the shortest likely most stable, respectively, among the four complexes. The results of distortion indices indicate that complexes 1–3 adopt Trigonal Bipyramid rather than Square Pyramid. If the coordinate bonds by small methanol or water are ignored, all the complexes belong to pseudo-tetrahedron (See Figure 2). The NBO partial charges of coordinate bond atoms seem insensitive to the impacts from different functional groups such tert-butyl, methyl, and chlorine.

### 3.2. Intramolecular Correlation Energy of Complexes and NBO-Based Perturbation Theory Energy Analysis

**Figure 4:**
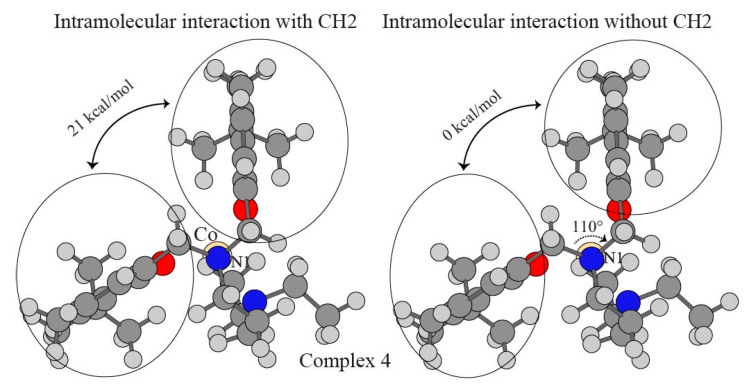
Intramolecular π–π within the complexes.

**Figure 5:**
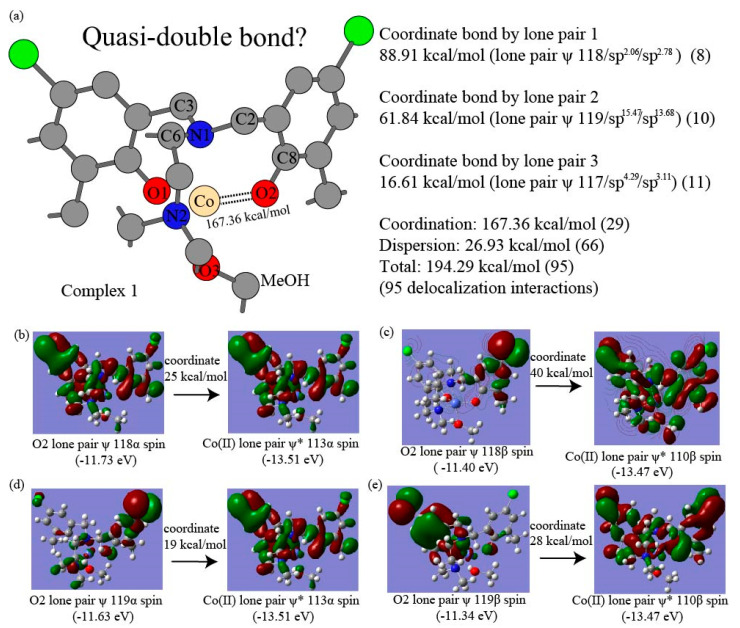
The delocalization interactions between donor atom O2 of ligand and central Co(II) ion in complex 1. (**a**) The Co(II)-O2 coordinate bond energies in complex 1; (**b**) The delocalization from O2 ψ lone pair 118α spin orbital to Co(II) ψ* lone pair 113α spin orbital; (**c**) The delocalization from O2 ψ lone pair 118β spin orbital to Co(II) ψ* lone pair 110β spin orbital; (**d**) The delocalization from O2 ψ lone pair 119α spin orbital to Co(II) ψ* lone pair 113α spin orbital; (**e**) The delocalization from O2 ψ lone pair 119β spin orbital to Co(II) ψ* lone pair 110β spin orbital (The ψ represents bonding orbitals and ψ* indicates antibonding orbitals).

**Figure 6:**
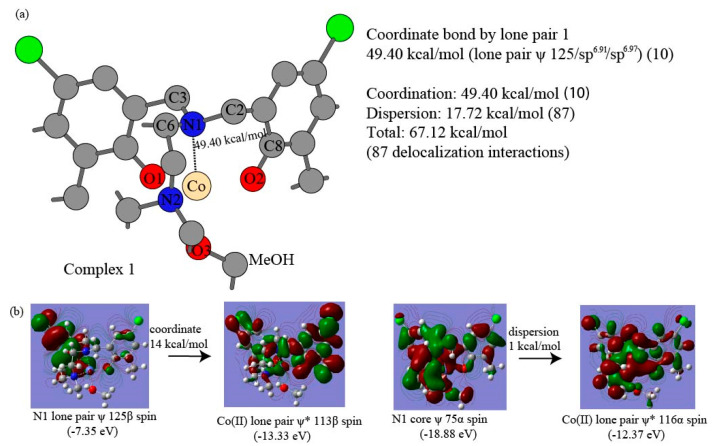
The delocalization interactions between donor atom N1 of ligand and central Co(II) ion in complex 1. (**a**) The Co(II)-N1 coordinate bond energies in complex 1. (**b**) The two out of 49 delocalization interactions between donor N1 and Co(II) ion (The ψ represents bonding orbitals and ψ* indicates antibonding orbitals).

**Figure 7:**
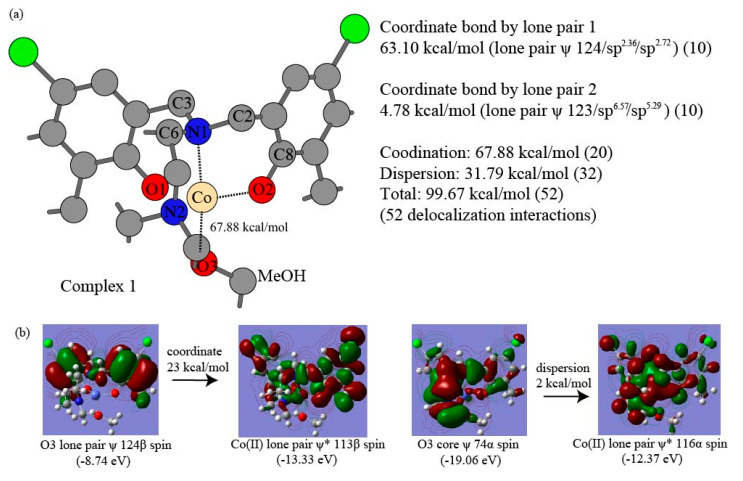
The delocalization interactions between donor atom O3 of methanol and central Co(II) ion in complex 1: (**a**) The Co(II)-O3 coordinate bond energies, (**b**) The two out of 52 delocalization interactions between donor O3 and Co(II) ion (The ψ represents bonding orbitals and ψ* indicates antibonding orbitals).

**Figure 8:**
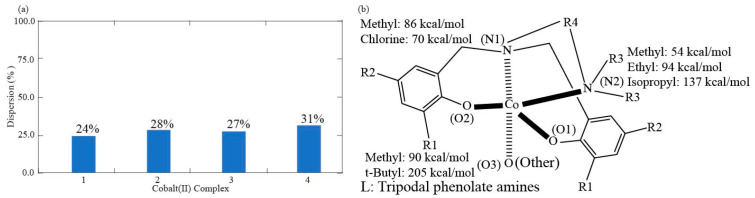
Percentages of dispersion between ligands and Co(II) and interaction contribution of different groups in the ligand. (**a**) Percentages of dispersion forces between ligand and Co(II) of four complexes; (**b**) The delocalization interactions contributed by groups in the rational design of ligands.

The major intramolecular interactions are evaluated using truncated fragments with basis set superposition correction (BSSE), solvation correction, and NBO-based perturbation theory energy analysis. The intramolecular π–π interaction energies between the dipodal phenolates are summarized for the most compact complex 4 of complexes in Table 6. The interacting pairs for dipodal branches of complex 4 are illustrated in Figure 4a,b. The two-way delocalization interactions include interactions from ligand donor atoms to central Co(II) and those from central Co(II) to ligand donor atoms to central Co(II) in the NBO-based perturbation theory energy analysis. The detailed Co(II)-O2 and Co(II)-N1 delocalization components from ligand donor atoms to central Co(II) ion in complex 1 are shown in Table 7. The representative delocalization interactions from ligand donor atoms O2, N1, and O3 to Co(II) are depicted in Figure 5, Figure 6, and Figure 7 for Co(II)-O2, Co(II)-N1, and Co(II)-O3, respectively. The delocalization components Co(II)-O2 and Co(II)-N1 from Co(II) to ligand donor atoms in complex 1 are depicted in Table 8. The two types of intramolecular interaction forces between ligand molecules and cobalt(II) ions are summarized for all four complexes in Table 9. The percentages of the dispersion force between ligand and central ions are depicted in Figure 8a. The delocalization interactions between methanol molecules or water and cobalt ions for the first three complexes are summarized in Table 10. Energetic contributions by functional groups methyl, tert-butyl, chlorine, ethyl, and isopropyl are summarized based on the Fock Matrix in NBO Basis (see Table 11 and Figure 8b).

The intramolecular π–π interaction was able to influence physical properties such as molecular fluorescence and magnetic properties [80,108,109,110]. The tripodal phenolate amines have two phenyl rings, and intramolecular π–π interaction is characterized in complex 4 with the most compact geometry based on the short bond length in Table 1. The inclusion of correlation plays a critical part in the evaluation of π–π interaction [111,112]. The correlation energy usually refers to the difference between true total energy and the Hartree–Fock limit. There are two major static and dynamic components in correlation energy. HF treats the many-body wavefunction as a single Slater determinant, while the calculation of the real total energy requires a combination of many Slater determinants. The approximation accounts for the lack of nondynamic correlation energy. The dynamical correlation refers to the lowering of real instantaneous coulomb repulsion by the assumption of all other electrons as an average distribution charge. HF already contains the static exchange-correlation between parallel spin electrons based on the anti-symmetry principle. Our results in Table 6 suggest that there is neither attraction nor repulsion associated with the moiety pair if the two CH_2_ directly bonded to the N1 atom are not included, which is described in Figure 4a. On the other hand, the inclusion of CH_2_ in the phenolate interaction yields a repulsive force of 20.97 kcal/mol. This implies that a certain extent of spatial tension happens between the two CH_2_ on the N1 atom even if the C-N1-C is 110°, which is shown in Figure 4b. It is worthwhile pointing out that the 110° C-N1-C angle is slightly larger than the 107° H-N-H in ammonia, and the smaller angle but larger size of CH_2_ than the H atom can rationalize the repulsive force in complex 4.

The Second Order Perturbation Theory Analysis of Fock Matrix in NBO Basis can provide insights into the intramolecular delocalization interactions within complex molecules. The metal ion coordination chemistry serves as a Lewis acid or electrophile, whereas ligand is treated as a Lewis base or nucleophile. The delocalization interactions due to the charge transfer from the ligand to the central cobalt ion usually involve bonding type orbitals of ligand and antibonding orbitals of central Co(II) ion. Weak opposite delocalization interaction also occurs because of the other way charge transfers from central cobalt ion to ligands and resultant delocalizations. Our NBO-based analyses show that the ligand provides donor oxygen atom lone pair bonding type orbitals (LP), oxygen core bonding type orbitals (CR), and O-C bonding orbital in the O-related delocalization, and the cobalt(II) ion offers lone pair antibonding orbitals (LP*) and Rydberg antibonding orbitals (RY*) accordingly. A coordinate bond is defined as a two-center covalent bond in which two electrons derive from the same nonmetal donor atom [113]. The coordinate bond in this work is described as delocalizations from LP orbitals of ligand donor oxygen or nitrogen atoms to the LP* orbitals of Co(II), and the rest of the types of delocalizations between the same ligand donor atoms and acceptor Co(II) are treated as the components of dispersion force. The dispersion force includes delocalization between LP orbitals and RY* orbitals, delocalization between O-C bonding orbitals and LP* orbitals, delocalization between core bonding orbitals (CR) and LP* orbitals, and delocalizations between O-C bonding orbitals and RY* orbitals. Unrestricted open shell calculations manage α spin orbitals and β spin orbitals separately for cobalt(II) ion, and each type of above delocalizations is also classified in terms of α and β spin orbitals accordingly. Table 7 shows that the coordinate bond has 10 delocalization interactions between O2 (for all labels of atoms, see Figure 2a) α spin lone pair bonding orbitals and Co(II) α spin lone pair antibonding orbitals, contributing 75.09 kcal/mol to the O2-Co(II) interaction in complex 1. Other 13 delocalization interactions that are responsible for 9.12 kcal/mol are considered the dispersion force from oxygen atom O2 to cobalt ion α spin orbitals. Similarly, 92.27 kcal/mol of coordinate bond force results from the 19 delocalization interactions between O2 β spin lone pair bonding orbitals of LP 117β, LP 118β and LP 119β and Co(II) β spin lone pair antibonding orbitals. The remaining 13 delocalization interactions contribute 9.10 kcal/mol, accounting for the dispersion force between O2 β spin bonding orbitals and Co(II) β spin antibonding orbitals. Figure 5 shows four major delocalization interactions between Co(II) and ligand donor oxygen atom O2. Figure 5b indicates 25 kcal/mol contributed by the delocalization from ligand O2 lone pair bonding orbital 118α to Co(II) lone pair antibonding orbital 113α, and Figure 5c exhibits 40 kcal/mol yielded by the delocalization from ligand oxygen atom O2 lone pair bonding orbital 118β to Co(II) lone pair antibonding orbital 110β. Additionally, Figure 5d exhibits 17 kcal/mol introduced by the delocalization from ligand O2 lone pair bonding orbital 118α to Co(II) lone pair antibonding orbital 114α of Co(II). Figure 5e shows 19 kcal/mol contributed by the delocalization from ligand O2 lone pair bonding orbital 119α to Co(II) lone pair antibonding orbital 113α, and Figure 5c exhibits 28 kcal/mol introduced by the delocalization from ligand O2 lone pair bonding orbital 119β to Co(II) lone pair antibonding orbital 110β. Interestingly, 29 delocalization interactions, which account for 167.36 kcal/mol of the O2-Co(II) coordinate bond, come from three lone pair bonding orbitals 117(117α + 117β), 118(118α + 118β), and 119(119α + 119β). The 11 delocalization interactions out of the 29 ones, which involve lone pair orbital 117, lead to 16.61 kcal/mol, implying it unlikely makes a significant part of the coordinate bond. The eight delocalization interactions resulting from lone pair orbital 118 provide 88.91 kcal/mol, whereas ten delocalization interactions enabled by lone pair orbital 119 give rise to 61.84 kcal/mol. Therefore, the lone pair orbital 118 and 119 probably form two separate coordinate bonds between donor O2 and Co(II). Furthermore, the O2 lone pair bonding orbitals exhibit quasi-σ bond features with hybridizations sp^2.06^ and sp^2.78^ for 118α and 118β, respectively, and the O2 lone pair bonding orbitals display a quasi-π bond character with essentially pure p-type hybridizations sp^15.47^ and sp^13.68^ for 119α and 119β, respectively. The slightly longer Co(II)-O coordinate bonds of 1.952 to 2.239 Å were studied [95], but bond types were not further characterized experimentally. Since we have no additional experimental evidence, the delocalization interactions of 167.36 kcal/mol between O2 LP orbitals to Co(II) LP* orbitals can be tentatively termed as an unusual coordinate quasi-double bond, which is illustrated in Figure 5a. The ratio of β spin coordinate bond over α spin coordinate bond is 1.2 for O2-Co(II), implying the unpaired electrons occupying the β spin bonding orbitals. The results collected in Table 7 and Figure 5a show that 26 non-coordinate bond delocalization interactions account for a dispersion force of 18.22 kcal/mol between O2 lone pair bonding orbitals and Co(II) lone pair antibonding orbitals. Similarly, Table 7 and Figure 6 display that 10 delocalization interactions originating from lone pair orbital 125 (125α + 125β) produce 49.10 kcal/mol for the coordinate bond force of N1-Co(II), and the rest of the 39 delocalization interactions provide 49.10 kcal/mol for dispersion force of N1-Co(II). The ratio of β spin coordinate bond over α spin coordinate bond is 1.5 for N1-Co(II), suggesting the unpaired electrons occurring in the β spin antibonding orbitals. It is worthwhile to notice that coordinate bond strength Co(II)-O2 is triple that of Co(II)-N1. Table 8 presents the opposite delocalization interactions due to charge transfer from Co(II) ion to ligand O2 and N1, all of which are considered dispersion forces between Co(II) ion and ligand donor atoms. There are 23 delocalization interactions between Co(II) ion α spin orbitals and O2 antibonding orbitals for 5.49 kcal/mol and 3.22 kcal/mol result from 17 delocalization interactions between Co(II) ion β spin orbitals and O2 antibonding orbitals. There are 26 delocalization interactions between Co(II) ion α spin orbitals and N1 antibonding orbitals for 1.97 kcal/mol and 1.51 kcal/mol result from 22 delocalization interactions between Co(II) ion β spin orbitals and N1 antibonding orbitals. Table 9 shows a summary of the coordinate bonds and dispersion forces between ligand and Co(II) ion for the four complexes. While averaged coordinate bond energies are 51.60 kcal/mol and 55.34 kcal/mol for Co(II)-N1 and Co(II)-N2, respectively, the Co(II)-O1 and Co(II)-O2 contribute averaged coordinate bond strengths of 149.24 kcal/mol and 154.56 kcal/mol, respectively. This consistently suggests the occurrences of uncommon coordinate double bonds in the four complexes. These findings are supported by the relatively short Co(II)-O bond length of (1.89–1.97 Å) in the four complexes illustrated in the previous section. The short coordinate bonds give rise to stronger ligand fields, which in turn enable larger splitting of the d-orbitals for higher energy but lower intensity of the d-d transition bands following Laporte election rules [114]. The UV-visible bands 485, 495, 553, and 541 nm with low absorptivity are consistent with the strong Co(II)-O coordinate bonds for the four complexes [77]. The charge transfer (CT) bands occur at 368–373 nm in cobalt (II) complexes [115]. The shoulder band wavelength (λ_max_) 380 nm for complexes 2 and 4 probably indicates the CT resulting from the oxygen LP to Co(II) LP* delocalization for Co(II)-O with a bond length of 1.89–1.95 Å in our previous UV/Vis spectral work [77]. The coordinate bond energies of Co(II) ion β spin orbitals are 1.2–1.6-fold of those from Co(II) ion α spin orbitals. The ligand-to-metal ion charge transfer is 11–12-fold of those from central ions to ligands, proposing the four complexes belong to ligand-to-metal charge transfer compounds [116]. The coordinate bonds constitute 76%, 72%, 73%, and 69% for complexes 1 to 4, respectively, and percentages of dispersion forces are 24%, 28%, 27%, and 31% in Figure 8a. Table 10 and Figure 7 show a summary of the coordinate bonds and dispersion forces between MeOH and Co(II) ion for the first three complexes. The Co(II)-O coordinate bonds provide binding energies of 67.88 kcal/mol, 63.89 kcal/mol, and 68.85 kcal/mol for complexes 1–3, respectively, and these bonds with small ligand molecules belong to regular coordinate bonds. The 10 delocalizations interactions stemmed from lone pair orbital 124 (124α + 124β) of donor O3 offer an interaction energy of 63.10 kcal/mol, meanwhile other 10 delocalization interactions led by lone pair orbital 123 (123α + 123β) of ligand donor atom O3 give rise to 4.78 kcal/mol. The coordinate bond strengths between MeOH and Co(II) ions are constitute 68%, 66%, and 69% of overall interaction between for complexes 1–3 respectively.

The Second Order Perturbation Theory Analysis of Fock Matrix in NBO Basis is also performed for functional groups methyl, tert-butyl, chlorine, ethyl, and isopropyl (see Table 11 and Figure 8b). While R1 methyl contributes 90 kcal/mol, the R1 tert-butyl provides 205 kcal/mol, which is almost double that of methyl. The R2 chlorine and methyl interact with the rest part of the molecule slightly differently and offer delocalization energy of 70 and 86 kcal/mol, respectively. The R3 groups isopropyl, ethyl, and methyl gave rise to 137 kcal/mol, 94 kcal/mol, and 55 kcal/mol, respectively, to the complexes through the N2 atom. Interestingly, the aromatic methyl group contributes 35 kcal/mol more than the amine methylation. Therefore, selections of proper functional groups at appropriate positions on tripodal tetradentate phenolate amines permit fine-tuning of the chemical and magnetic properties of SMM candidates.

### 3.3. Effect of Correlation Dispersion Force and Magnetic Axial and Transverse ZFS

**Figure 9:**
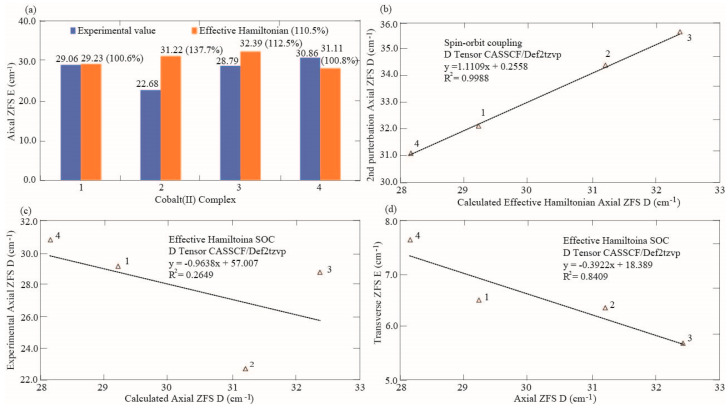
Comparisons of theoretical magnetic susceptibility parameters with experimental values. (**a**) Comparison between the second perturbation axial ZFS D and experimental counterpart. (**b**) Correlation between the effective Hamiltonian axial ZFS D and second perturbation counterpart. (**c**) Correlation between the effective Hamiltonian axial ZFS D tensor and the experimental axial ZFS D index. (**d**) Correlation between the effective Hamiltonian axial ZFS D results and the effective Hamiltonian transverse ZFS E values.

**Figure 10:**
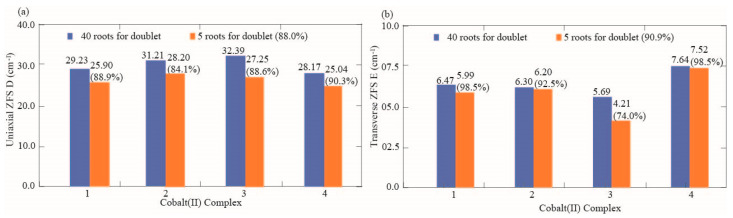
Comparison between theoretical magnetic susceptibility. (**a**) Comparison of the axial ZFS D values between the calculations of 40 doublet roots and those of 5 doublet roots. (**b**) Comparison of the transverse ZFS E values between the calculations, including 40 doublet roots and those specifying 5 doublet roots.

Magnetic susceptibility parameters are simulated using the CASSCF method. The direct current (DC) induced equilibrium magnetic behaviors are focused in this work, and the alternating current (AC) involved dynamic magnetizations, which had been the topic in previous efforts [77]. There are 10 quartet roots and 40 doublet roots specified in the CASSCF simulations of the complexes. Contributions to ZFS D from 10 quartet roots and 40 doublet roots in the CASSCF calculation are listed in Table 12. The single point energies from Gaussian-16 calculations are compared with the lowest energy of the quartet roots from the CASSCF simulation in Table 13. The calculation results of axial D, transverse ZFS E, isotropic g factors, and the experimental spin Hamiltonian (SH) magnetic susceptibility ZFS D indices and g factors, which are extracted by fitting both the magnetic susceptibility *χ_M_T* vs. temperature *T* and magnetization [92], are shown in Table 14. Comparisons of the theoretical magnetic susceptibility parameters and experimental values are represented in Figure 9. The first- and second order perturbation to the effective Hamiltonian is reported to be significant for excitation from the ground configuration and lower excitation levels [117]. Comparisons are made between the results of calculations using 40 doublet roots and those using 5 doublets for axial zero-field splitting (ZFS) D and transverse ZFS E from the second order perturbation and effective Hamiltonian calculation of spin–orbit coupling (SOC) in Table 15. Comparisons between theoretical magnetic susceptibility parameters and experimental values are described in Figure 10. Results in Table 12 indicate that the 40 doublet roots contribute 24%, 23%, 23%, and 23% to the ZFS D tensor for four complexes respectively. The doublet and quartet spin states both occur in the complexes, and the contributions to the overall electronic and magnetic properties depend on variety of factors such as, ligand field strength and spin–orbit coupling [118]. The effects of the above factors will be further investigated using the ab initio ligand field theory (AILFT) in future work [119]. Table 13 shows that the lowest energies of quartet roots from CASSCF calculations—100.38 kcal/mol, 100.54 kcal/mol, 105.48 kcal/mol, and 99.96 kcal/mol—are lower than those of HF energies for the complexes. Furthermore the missing correlation energies of −2632.19 kcal/mol (−10.40 kcal/mol per electron), −3008.97 kcal/mol (−10.56 kcal/mol per electron), −3129.20 kcal/mol (−11.30 kcal/mol per electron), and −3273.84 kcal/mol (−11.25 kcal/mol per electron) for the four complexes respectively may prevent accurate CASSCF evaluations of energy levels of 40 doublet roots and 10 quartet roots as well as the magnetic susceptibility parameters. Table 14 demonstrates the spin–orbit coupling dominates in axial D tensor with 32.118 cm^−1^, 34.468 cm^−1^, 35.724 cm^−1^, and 31.106 cm^−1^ for complexes 1 to 4, respectively, whereas the corresponding spin–spin contribution of −0.028 cm^−1^, 0.031 cm^−1^, −0.031 cm^−1^, and 0.024 cm^−1^, which are almost negligible to the second order perturbation D tensor. Similar trends about spin–spin coupling occur to the second order perturbation calculations of the transverse E tensor and the effective Hamiltonian simulation of both axial D tensor and transverse E tensor. The axial D tensors are 5-, 5-, 6-, and 4-fold, respectively, for complexes 1–4 of transverse E tensors in both the second order perturbation and effective Hamiltonian calculations. The experimental values for axial D tensors and g factors are listed for the complexes in Table 14. The experimental D tensor values are 29.060 cm^−1^, 22.680 cm^−1^, 28.790 cm^−1^, and 30.860 cm^−1^ for complexes 1 to 4, respectively, and the second order perturbation calculations provide D tensor values which are 110.4%, 151.8%, 124.0%, and 100.7% of the experimental counterparts, respectively. Table 14 indicates that the average of the theoretical second order perturbation D tensors is 121.8% of the experimental results, and the effective Hamiltonian computation presents an average 110.5% of the experimental value which is also described in Figure 9a. While Figure 9b presents a perfect correlation with R^2^ of 0.9988 between the theoretical Effective Hamiltonian and the second order perturbation ZFS D tensors, a poor inverse correlation of R^2^ 0.2648 occurs between theoretical Effective Hamiltonian and experimental ZFS D tensors (Figure 9c). The low correlation may be attributed to a lack of major correlation energy in the CASSCF calculations. It is known that combination of strong correlations and spin–orbit coupling can lead to highly anisotropic exchange interactions [9,60,120,121]. The lack of capturing the dynamic correlation energy leads to less accurate energy calculations due to failure to account for the instantaneous interaction among electrons [122]. Figure 9d shows that the axial tensor D exhibits an inverse correlation with respect to the transverse E tensor with an R^2^ value of 0.8409. Our computations yield ZFS D parameters 28–31 cm^−1^, which are close to 35–42 cm^−1^ by Mitsuhashi et al. [64]. The Lande or Gyromagnetic ratio (g-factor) is defined as the ratio of a molecule’s magnetic moment to its angular moment [123], and its magnitude reflects how strongly the spin and orbital of a molecule are modulated by an applied magnetic field. The electronic structure of the phenolate donor in Co(II) complexes was shown to exert strong effects on the *g*-factors of the compounds [124]. The theoretical and experimental values of g-factors are listed for the complexes in Table 14, and the comparisons suggest that theoretical g factors agree well with experimental results, with an average percent of 98.10% of theoretical values over experimental results. The simulation results of g-factors (g_x_ = 2.028 to 2.052, g_y_ = 2.221 to 2.251, and g_z_ = 2.372 to 2.387) agree well with those (g_x_ = 2.11 to 2.15, g_y_ = 2.11 to 2.15, and g_z_ = 2.48 to 2.56) of previous works [64]. Specifying 5 doublet roots rather than 40 doublet roots could allow quick evaluations of large number of complex candidates. The result in Table 15 suggests the simulation including 5 doublet roots instead of 40 doublet roots can cover 87.88% of axial D tensor values calculated using second order perturbation, and 87.98% in the effective Hamiltonian calculation. Similarly, the second order perturbation method calculation with 5 doublet roots specified gives 90.00% transverse E tensor obtained the second order perturbation method where 40 doublet roots are considered (Seen in Figure 10a), and the percentage turns to 90.88% in the effective Hamiltonian computation which is exemplified in Figure 10b. The Co(II) complex with high anisotropy of magnetic sustability D of −140 ± 30 cm^–1^ was reported [125]. In the future, we hope to establish an accurate structural model for Co(II) complexes, which will help the development of Co(II)-tripodal phenolate amines or other ligands of high magnetic sustainability.

## 4. Discussion

Crystal packing in a crystal lattice can significantly impact the magnetic properties of Co(II) complexes. Density functional theory (DFT) based molecular dynamics will be performed to investigate the effect of crystal packing on forces, stresses, and other properties of the SMMs. The inconsistencies between theoretical and experimental axial ZFS D for the complexes probably result from an inappropriate treatment of correlation energy in the CASSCF calculation.

## 5. Conclusions

The four Co(II) complexes were characterized using MP2 and CASSCF methods. The short Co(II)-O (ligand) coordinate bonds with lengths of 1.89 Å to 1.97 Å are identified as unusual coordinate quasi-double bonds in the optimized geometries. The strengths of Co(II)-O coordination bonds range from 142.01 kcal/mol to 167.36 kcal/mol, whereas Co(II)-N bond energies only have 48.93 kcal/mol to 60.11 kcal/mol. The delocalization energies of Co(II)-O coordination bonds of ligand are two-fold of those for Co(II)-O coordinate bonds of methanol, which are 67.88 kcal/mol, 63.89 kcal/mol, and 68.85 kcal/mol for complex 1 to 3 separately. The lone pair orbital 118 and 119 of ligand donor O2 probably form two separate coordinate bonds between donor O2 and Co(II). The O2 lone pair bonding orbitals show quasi-σ bond feature with hybridizations sp^2.06^ and sp^2.78^ for 118α and 118β, respectively, and the O2 lone pair bonding orbitals demonstrate a quasi-π bond character with essentially pure p-type hybridizations sp^15.47^ and sp^13.68^ for 119α and 119β, respectively. The delocalization interactions of 167.36 kcal/mol between O2 lone pair bonding orbitals to Co(II) lone pair antibonding orbitals can be tentatively termed as an unusual coordinate quasi-double bond due to the absence of direct supporting experimental results. The dispersion forces constitute 24%, 28%, 27%, and 31% of the ligand–central ion interaction. The MP2 calculation indicated the π–π interaction does not occur between the two phenolate moieties, but a repulsive force of 20.97 kcal/mol exists between two CH_2_ groups bonded to N1 atoms. The delocalization energy for the ligand to the central metal ion is 11-fold that of the opposite central Co(II) to ligands, suggesting that the complexes are dominated by the ligand-to-metal charge transfer (LMCT). The tert-butyl group on the phenyl ring provides the highest 204.74 kcal/mol to intramolecular interaction energy among methyl, ethyl, chloro, and tert-butyl groups. This work shows that spin–orbit coupling constitutes the major component of both axial and transverse ZFS. The axial ZFS D has three-fold of the transverse ZFS E, and the axial ZFS D is inversely correlated with the transverse ZFS values in the complexes. Our results propose that computations specifying 5 doublet roots can cover 85% of D and E numerical values from the calculations, including 40 doublet roots. The Effect Hamiltonian method generates more accurate D and E numerical values than those from the second order perturbation approach. This work suggests that the bulky tert-butyl and the longer spacer [CH_2_]_2_ can tune dispersion forces in the Co(II) complexes to boost large magnetic susceptibility. Our results imply the need to employ an MP2 type of complete active space strategy for the simulation of SMM materials. Our calculations suggest that the combination of bulky electron donating groups and the linker [CH_2_]_2_ can be utilized to pursue Co(II) compounds of promising magnetic properties and Co(II) porphyrin fullerene conjugate systems for CO_2_ electroreduction.

## Figures and Tables

**Table 1:** Comparison of calculated and actual values of the spin operator of four Co(II) complexes.

Complex	1	2	3	4
Overall atoms	57	69	78	84
Total Mass	486.30	542.41	515.60	539.67
Before annihilation	3.7575	3.7575	3.7575	3.7575
After annihilation	3.75	3.75	3.75	3.75
Theoretical value	3.75	3.75	3.75	3.75

**Table 2:** Coordination bond lengths for the optimized structures of four Co(II) complexes (Å).

Complex	Mean	1	2	3	4
Co(II)-N1	2.15	2.19	2.17	2.16	2.06
Co(II)-N2	2.15	2.12	2.20	2.11	2.06
Co(II)-O1	1.94	1.95	1.95	1.97	1.89
Co(II)-O2	1.93	1.94	1.94	1.93	1.90
Co(II)-O3	2.12 *	2.11	2.13	2.13	

* The averages are calculated for the three available values.

**Table 3:** The LG-M-LG bond angles of four Co(II) complexes (degree°).

Complex	Mean	1	2	3	4
N1-Co(II)-O2	91	92	91	91	91
N1-Co(II)-O1	94	91	92	93	99
N1-Co(II)-N2	88	82	83	84	101
N2-Co(II)-O1	112	107	108	113	118
N2-Co(II)-O2	123	130	133	120	107
O1-Co(II)-O2	124	123	119	127	126
N1-Co(II)-O3	175	175	179	171	
N2-Co(II)-O3	94 *	93	96	93	
O1-Co(II)-O3	91 *	90	88	96	
O2-Co(II)-O3	88 *	91	89	83	

* The averages are calculated for the three available values.

**Table 4:** Geometric index based on L-M-L bond angles for four Co(II) complexes.

Complex	1	2	3	4
τ_4_	0.75623	0.76332	0.79877	0.82004
τ_5_	0.75015	0.76682	0.73348	

**Table 5:** The Natural Bond Orbital (NBO) partial charge for the donor atoms with MP2/DeF2SVP using Gaussian 16 Revision B.01.

Atom	Mean	1	2	3	4
Co(II)	2.18	2.17	2.18	2.17	2.19
N1	−0.34	−0.35	−0.33	−0.34	−0.35
N2	−0.34	−0.32	−0.35	−0.33	−0.34
O1	−0.46	−0.40	−0.48	−0.48	−0.48
O2	−0.46	−0.41	−0.48	−0.48	−0.48
O3	−0.43 *	−0.42	−0.41	−0.47	

* The averages are calculated for the three available values.

**Table 6:** Intramolecular π–π force between the dipodal phenolates (restricted open shell MP2).

pi–pi (no CH_2_) ^a^	ΔE_gas_^b^	ΔΔE_gas_^BSSE c^	ΔΔE_solv_^d^	ΔE
pi–pi (with CH_2_)	70.33	5.16	−54.52	20.97
pi–pi (no CH_2_)	−4.23	3.35	0.98	0.10

^a^ All methods used the aug-cc-pVDZ basis set. ^b^ ΔE_gas_ = E_dimer(gas)_ − E_monomer,a(gas)_ − E_monomer,b(gas)_. ^c^ ΔΔE_gas_^BSSE^ denotes the counterpoise correction for the basis set superposition error. ^d^ ΔΔE_solv_ = (ΔE _dimer(solv)_ − ΔE _monomer,a(solv)_ − ΔE _monomer,b(solv)_) − ΔE_gas_, where ΔE_solv_ denotes solvation free energy.

**Table 7:** Delocalization energy from ligands to Co(II) ion in the Second Order Perturbation Theory Analysis of Fock Matrix in NBO Basis for O2-Co(II) and N1-Co(II) of complex 1 (The relevant atoms are labeled in Figure 2. Unit: kcal/mol), LP represents lone pair bonding orbitals, LP* indicates lone pair antibonding orbitals, RY denotes the Rydberg orbital, RY* stands for antibonding Rydberg orbitals, CR is the abbreviation of inner core Co(II) orbitals, and BD represents bonding orbitals.

O2-Co(II)					N1-Co(II)				
Donor	Orbital	Acceptor	Orbital	Energy	Donor	Orbital	Acceptor	Orbital	Energy
O2	LP 117α	Co(II)	LP* 113α	3.70	N1	LP 125α	Co(II)	LP* 113α	3.42
O2	LP 117α	Co(II)	LP* 114α	0.78	N1	LP 125α	Co(II)	LP* 114α	0.15
O2	LP 117α	Co(II)	LP* 115α	0.84	N1	LP 125α	Co(II)	LP* 115α	3.82
O2	LP 117α	Co(II)	LP* 116α	1.13	N1	LP 125α	Co(II)	LP* 116α	12.55
O2	LP 118α	Co(II)	LP* 113α	25.26					
O2	LP 118α	Co(II)	LP* 114α	16.95					
O2	LP 119α	Co(II)	LP* 113α	18.59					
O2	LP 119α	Co(II)	LP* 114α	4.29					
O2	LP 119α	Co(II)	LP* 115α	3.40					
O2	LP 119α	Co(II)	LP* 116α	0.15					
Coordinate			10	75.09				4	19.94
O2	LP 117α	Co(II)	RY* 133α	0.03	N1	LP 125α	Co(II)	RY* 134α	0.06
O2	LP 117α	Co(II)	RY* 137α	0.05	N1-C2	BD 5α	Co(II)	LP* 113α	0.07
O2	LP 118α	Co(II)	RY* 133α	0.05	N1-C2	BD 5α	Co(II)	LP* 114α	0.38
O2	LP 118α	Co(II)	RY* 134α	0.07	N1-C2	BD 5α	Co(II)	LP* 115α	0.61
O2	LP 119α	Co(II)	RY* 133α	0.20	N1-C2	BD 5α	Co(II)	LP* 116α	0.79
O2	LP 119α	Co(II)	RY* 135α	0.05	N1-C3	BD 6α	Co(II)	LP* 113α	0.04
O2-C8	BD 1α	Co(II)	LP* 113α	2.97	N1-C3	BD 6α	Co(II)	LP* 114α	0.37
O2-C8	BD 1α	Co(II)	LP* 114α	1.76	N1-C3	BD 6α	Co(II)	LP* 115α	0.53
O2-C8	BD 1α	Co(II)	LP* 115α	0.12	N1-C3	BD 6α	Co(II)	LP* 116α	0.71
O2	CR 72α	Co(II)	LP* 113α	2.34	N1-C6	BD 7α	Co(II)	LP* 113α	0.03
O2	CR 72α	Co(II)	LP* 114α	1.35	N1-C6	BD 7α	Co(II)	LP* 114α	0.34
O2	CR 72α	Co(II)	LP* 115α	0.09	N1-C6	BD 7α	Co(II)	LP* 115α	0.59
O2-C8	BD 1α	Co(II)	RY* 133α	0.04	N1-C6	BD 7α	Co(II)	LP* 116α	0.77
					N1	CR 75α	Co(II)	LP* 113α	0.41
					N1	CR 75α	Co(II)	LP* 114α	0.03
					N1	CR 75α	Co(II)	LP* 115α	0.34
					N1	CR 75α	Co(II)	LP* 116α	0.98
					N1-C3	BD 6α	Co(II)	RY* 133α	0.04
					N1-C6	BD 7α	Co(II)	RY* 133α	0.03
Dispersion			13	9.12				19	7.12
O2	LP 117β	Co(II)	LP* 110β	5.87	N1	LP 125β	Co(II)	LP* 110β	1.44
O2	LP 117β	Co(II)	LP* 111β	0.39	N1	LP 125β	Co(II)	LP* 111β	0.26
O2	LP 117β	Co(II)	LP* 112β	0.28	N1	LP 125β	Co(II)	LP* 112β	4.82
O2	LP 117β	Co(II)	LP* 113β	1.53	N1	LP 125β	Co(II)	LP* 113β	13.76
O2	LP 117β	Co(II)	LP* 114β	0.29	N1	LP 125β	Co(II)	LP* 114β	9.05
O2	LP 117β	Co(II)	LP* 115β	0.72	N1	LP 125β	Co(II)	LP* 116β	0.13
O2	LP 117β	Co(II)	LP* 116β	1.08					
O2	LP 118β	Co(II)	LP* 110β	35.90					
O2	LP 118β	Co(II)	LP* 111β	7.60					
O2	LP 118β	Co(II)	LP* 112β	0.11					
O2	LP 118β	Co(II)	LP* 114β	0.50					
O2	LP 118β	Co(II)	LP* 115β	1.88					
O2	LP 118β	Co(II)	LP* 116β	0.71					
O2	LP 119β	Co(II)	LP* 110β	28.07					
O2	LP 119β	Co(II)	LP* 111β	2.27					
O2	LP 119β	Co(II)	LP* 112β	2.13					
O2	LP 119β	Co(II)	LP* 114β	0.59					
O2	LP 119β	Co(II)	LP* 115β	1.71					
O2	LP 119β	Co(II)	LP* 116β	0.64					
Coordinate			19	92.27				6	29.46
O2	LP 117β	Co(II)	RY* 137β	0.04	N1-C3	BD 6β	Co(II)	LP* 133β	0.03
O2	LP 119β	Co(II)	RY* 133β	0.20	N1-C6	BD 5β	Co(II)	LP* 110β	0.41
O2-C8	BD 1β	Co(II)	LP* 110β	3.91	N1-C6	BD 5β	Co(II)	LP* 111β	0.12
O2-C8	BD 1β	Co(II)	LP* 111β	0.69	N1-C6	BD 5β	Co(II)	LP* 112β	0.48
O2-C8	BD 1β	Co(II)	LP* 112β	0.11	N1-C6	BD 5β	Co(II)	LP* 113β	0.77
O2-C8	BD 1β	Co(II)	LP* 114β	0.06	N1-C6	BD 5β	Co(II)	LP* 114β	0.09
O2-C8	BD 1β	Co(II)	LP* 116β	0.28	N1-C3	BD 6β	Co(II)	LP* 110β	0.34
O2	CR 72β	Co(II)	LP* 110β	2.94	N1-C3	BD 6β	Co(II)	LP* 111β	0.10
O2	CR 72β	Co(II)	LP* 111β	0.42	N1-C3	BD 6β	Co(II)	LP* 112β	0.45
O2	CR 72β	Co(II)	LP* 112β	0.07	N1-C3	BD 6β	Co(II)	LP* 113β	0.72
O2	CR 72β	Co(II)	LP* 114β	0.06	N1-C3	BD 6β	Co(II)	LP* 114β	0.10
O2	CR 72β	Co(II)	LP* 115β	0.03	N1-C2	BD 7β	Co(II)	LP* 110β	0.40
O2	CR 72β	Co(II)	LP* 116β	0.29	N1-C2	BD 7β	Co(II)	LP* 111β	0.07
					N1-C2	BD 7β	Co(II)	LP* 112β	0.46
					N1-C2	BD 7β	Co(II)	LP* 113β	0.75
					N1-C2	BD 7β	Co(II)	LP* 114β	0.08
					N1	CR 75β	Co(II)	LP* 110β	0.32
					N1	CR 75β	Co(II)	LP* 111β	0.08
					N1	CR 75β	Co(II)	LP* 112β	0.39
					N1	CR 75β	Co(II)	LP* 113β	0.96
Dispersion			13	9.10				20	7.12
O2 LP 117-Co(II)			11	16.61	N1 LP 125-Co(II)			10	49.40
O2 LP 118-Co(II)			8	88.91					
O2 LP 119-Co(II)			10	61.84					
Co(II) β/α))				1.2					1.5
Coordinate Tot			29	167.36				10	49.40
Dispersion Tot			26	18.22				39	14.24

**Table 8:** Delocalization energy from Co(II) ion to ligands in the Second Order Perturbation Theory Analysis of Fock Matrix in NBO Basis for O2-Co(II) and N1-Co(II) of complex 1 (Unit: kcal/mol). LP represents lone pair bonding orbitals, LP* indicates lone pair antibonding orbitals, RY denotes the bonding Rydberg orbital, RY* stands for antibonding Rydberg orbitals, CR is the abbreviation of inner Co(II) orbitals, BD represents bonding orbitals, and BD* inciates antibonding orbitals.

Co(II)-O2					Co(II)-N1				
Donor	Orbital	Acceptor	Orbital	Energy	Donor	Orbital	Acceptor	Orbital	Energy
Co(II)	CR/LP/LP*	O2	BD*/RY*		Co(II)	CR/LP/LP*	N1	BD*/RY*	
	α		23	5.49				26	1.97
	β		17	3.22				22	1.51
Dispersion Tot			40	8.71				48	3.48

**Table 9:** Summary of the delocalization energy of Second Order Perturbation Theory Analysis of Fock Matrix in NBO Basis for the four complexes (Unit: kcal/mol).

Complex Number of Atoms		1	2	3	4
		57	69	78	84
Donor	Acceptor				
N1 LP	Co(II) α spin LP*	19.94	20.39	19.99	22.37
N2 LP	Co(II) α spin LP*	23.37	19.78	21.68	24.61
O1 LP	Co(II) α spin LP*	75.09	63.84	67.33	68.20
O2 LP	Co(II) α spin LP*	66.10	62.49	66.65	69.21
Ligand	Co(II) α other	45.21	53.90	51.92	69.60
N1 LP	Co(II) β spin LP*	29.46	28.54	29.73	35.99
N2 LP	Co(II) β spin LP*	32.34	30.00	34.08	35.50
O1 LP	Co(II) β spin LP*	92.27	80.36	83.66	87.47
O2 LP	Co(II) β spin LP*	82.74	79.52	82.59	87.66
Ligand	Co(II) β other	44.76	54.22	52.28	71.38
Co(II) α spin other	N1 other	1.97	1.98	2.37	2.82
Co(II) α spin other	N2 other	2.95	2.17	3.16	3.27
Co(II) α spin other	O1 other	5.49	5.08	5.52	6.12
Co(II) α spin other	O2 other	5.30	5.61	5.20	5.75
Co(II) α spin other	other atoms	7.47	10.41	10.87	9.43
Co(II) β spin other	N1 other	1.51	1.13	1.58	1.95
Co(II) β spin other	N2 other	1.99	1.93	1.9	1.95
Co(II) β spin other	O1 other	3.22	3.04	2.92	3.19
Co(II) β spin other	O2 other	3.35	3.35	2.63	3.13
Co(II) β spin other	Other atoms	8.01	10.54	10.51	12.61
N1-Co(II) β/α	1.5	1.5	1.4	1.5	1.6
N2-Co(II) β/α	1.5	1.4	1.5	1.6	1.4
O1-Co(II) β/α	1.3	1.2	1.3	1.2	1.3
O2-Co(II) β/α	1.3	1.3	1.3	1.2	1.3
LP1 N1-Co(II)	(Mean) 51.60	49.40	48.93	49.72	58.36
LP1 O2-Co(II)	(Mean) 14.55	16.61	15.38	13.13	13.08
LP2 O2-Co(II)	(Mean) 80.73	88.91	85.43	64.87	83.71
LP3 O2-Co(II)	(Mean) 59.28	61.84	43.39	72.99	58.88
N1-Co(II)	(Mean) 51.60	49.40	48.93	49.72	58.36
N2-Co(II)	(Mean) 55.34	55.71	49.78	55.76	60.11
O1-Co(II)	(Mean) 154.56	167.36	144.20	150.99	155.67
O2-Co(II)	(Mean) 149.24	148.84	142.01	149.24	156.87
L-M coordination	410.74	421.31	384.92	405.71	431.01
L-M dispersion	110.82	89.97	108.12	104.2	140.98
L-M interaction	521.56	511.28	493.04	509.91	571.99
M-L dispersion	45.85	41.26	45.24	46.66	50.22
L-M/M-L ratio	11.25	12	11	11	11
Dispersion	156.66	131.23	153.36	150.86	191.20
Total interaction	567.40	552.54	538.28	556.57	622.21
coordination %	72.5	76%	72%	73%	69%
Dispersion %	27.5	24%	28%	27%	31%

**Table 10:** Summary of the delocalization energy of Second Order Perturbation Theory Analysis of Fock Matrix in NBO Basis for methanol-involved coordinations in three complexes (Unit: kcal/mol).

Complex Number of Atoms		1	2	3	4
		57	69	78	84
Donor	Acceptor				
O3 LP	Co(II) α spin LP*	30.18	28.52	30.76	
MeOH	Co(II) α other	10.91	12.04	13.27	
O3 LP	Co(II) β spin LP*	37.70	35.37	38.09	
MeOH	Co(II) β other	11.11	12.22	13.26	
Co(II) α spin other	MeOH	4.87	4.57	2.59	
Co(II) β spin other	MeOH	4.75	4.34	2.40	
L-M O3-Co(II) β/α	(Mean) 1.2	1.2	1.2	1.2	
LP(ψ 124)-M O3-Co(II)	62.17	63.10	59.18	64.24	
LP(ψ 123)-M O3-Co(II)	4.70	4.78	4.71	4.61	
L-M O3-Co(II)	66.87	67.88	63.89	68.85	
L-M dispersion	24.27	22.02	24.26	26.53	
L-M interaction	91.14	89.90	88.15	95.38	
M-L dispersion	7.84	9.62	8.91	4.99	
L-M/M-L ratio	12.67	9	10	19	
Dispersion	32.45	31.79	34.17	31.40	
Total interaction	98.98	99.52	97.06	100.37	
coordination %	67.7	68%	66%	69%	
Dispersion %	32.3	32%	34%	31%	

**Table 11:** Summary of the delocalization energy of Second Order Perturbation Theory Analysis of Fock Matrix in NBO Basis for functional-groups-involved interactions in three complexes (Unit: kcal/mol).

Complex	1	2	3	4
R1	89.93	89.89	205.43	204.50
R2	70.17	70.15	86.16	85.13
R3	55.39	136.94	54.33	94.47
Ligand	2956.99	3056.88	2238.29	3857.57

**Table 12:** The contributions of ZFS D from 40 doublet roots and 10 quartet roots in the second perturbation computation.

Spin	Root	Complex 1	Complex 2	Complex 3	Complex 4
3/2	0	0.000	0.000	0.000	0.000
3/2	1	7.539	7.372	6.275	14.511
3/2	2	0.504	2.185	0.690	1.884
3/2	3	6.591	6.869	9.226	2.736
3/2	4	9.505	9.923	10.878	4.597
3/2	5	0.091	0.074	0.058	0.071
3/2	6	0.046	0.034	0.026	0.007
3/2	7	0.090	0.063	0.103	0.005
3/2	8	0.001	0.038	0.000	0.008
3/2	9	0.075	0.021	0.089	0.001
3/2	Tot	24.442	26.579	27.345	23.82
1/2	0	−0.226	−0.191	0.681	2.610
1/2	1	0.464	0.334	−0.044	0.166
1/2	2	3.412	3.500	2.410	0.216
1/2	3	−0.011	0.002	0.011	0.381
1/2	4	−0.248	0.013	−0.828	−0.008
1/2	5	−0.096	−0.055	−0.128	−0.031
1/2	6	−0.732	−0.671	−0.034	−1.331
1/2	7	0.097	0.286	8.538	7.684
1/2	8	6.237	5.737	−1.118	−1.038
1/2	9	−0.025	−0.026	−0.003	−0.231
1/2	10	0.000	0.012	−0.003	−0.266
1/2	11	−0.009	−0.008	−0.047	0.029
1/2	12	−0.086	−0.139	−0.055	0.028
1/2	13	−0.149	−0.111	−0.010	−0.210
1/2	14	−0.142	−0.075	−0.167	−0.071
1/2	15	−0.168	−0.166	−0.165	−0.017
1/2	16	0.005	−0.036	−0.018	−0.555
1/2	17	−0.105	−0.198	−0.112	−0.180
1/2	18	−0.156	−0.095	−0.178	0.097
1/2	19	−0.000	−0.141	0.115	−0.035
1/2	20	0.116	0.258	0.078	0.068
1/2	21	0.026	0.002	0.001	0.161
1/2	22	0.748	0.907	0.776	0.634
1/2	23	−0.432	−0.259	−0.107	−0.343
1/2	24	−0.026	−0.189	−0.258	−0.200
1/2	25	−0.165	−0.169	−0.111	−0.076
1/2	26	−0.214	−0.197	−0.311	−0.082
1/2	27	−0.266	−0.283	−0.340	−0.096
1/2	28	−0.066	−0.047	−0.107	−0.010
1/2	29	−0.125	−0.059	−0.105	0.045
1/2	30	−0.050	−0.072	−0.035	−0.044
1/2	31	−0.051	−0.058	−0.054	−0.044
1/2	32	0.006	−0.033	−0.070	−0.054
1/2	33	−0.038	−0.039	0.005	−0.020
1/2	34	−0.000	−0.001	0.007	−0.020
1/2	35	0.045	0.043	0.066	0.014
1/2	36	0.020	0.004	0.001	−0.000
1/2	37	−0.004	−0.003	−0.001	−0.001
1/2	38	−0.003	−0.004	−0.003	−0.004
1/2	39	0.093	0.119	0.100	0.124
1/2	Tot	7.676	7.892	8.377	7.290
Tot		32.118	34.471	35.722	31.11
3/2		76%	77%	77%	77%
1/2		24%	23%	23%	23%

**Table 13:** Calculated MP2, HF energies, and major correlation energies with MP2/Def2SVP method using Gaussian 16 Revision B.01.

Complex	1	2	3	4
Lowest CASSCF	−3444.3676637	−3600.3760521	−2799.8676682	−2840.8954558
HF(Hartree)	−3444.2077057	−3600.2158344	−2799.6995722	−2840.7361725
MP2(Hartree)	−3448.4023597	−3605.0109351	−2804.6862648	−2845.9533635
CASSCF-HF (kcal/mol)	−100.38	−100.54	−105.48	−99.96
MP2-HF (kcal/mol)	−2632.19	−3008.97	−3129.20	−3273.84
Number of electrons	253	285	277	291
MP2-HF (kcal/mol)/e	−10.40	−10.56	−11.30	−11.25
CASSCF-HF (kcal/mol)	−100.38	−100.54	−105.48	−99.96

**Table 14:** The axial zero-field splitting (ZFS)(D) and transverse ZFS(E) from spin–spin coupling (SSC), second order spin–orbit coupling (SOC), and effective Hamiltonian with CASSCF/Def2SVP method using ORCA package (version 5.01). D-tensor spin–orbit coupling is calculated using Pederson–Khanna (PK) algorithm (Unit: cm ^−1^).

Complex	1	2	3	4
Number of atoms	57	69	78	84
2nd order SOC D	32.118	34.468	35.724	31.106
SSC D	−0.028	0.031	−0.031	0.024
2nd order D	32.090	34.437	35.693	31.082
2nd order SOC E	6.837	6.687	5.980	8.125
SSC E	−0.007	0.009	−0.008	0.004
2nd order E	6.830	6.696	5.972	8.121
2nd order D/E	5	5	6	4
Effective Hamiltonian D	29.233	31.220	32.392	28.173
Effective Hamiltonian E	6.472	6.300	5.689	7.637
Effective Hamiltonian D/E	5	5	6	4
Experimental D based on (χ_M_)	29.060	22.680	28.790	30.860
2nd order perturbation D (%)	110.4	151.8	124.0	100.7
Effective Hamiltonian D (%)	100.6	137.7	112.5	91.3
Theoretical iso g factor	2.216	2.225	2.219	2.220
Theoretical g_x_ factor	2.046	2.040	2.028	2.052
Theoretical g_y_ factor	2.229	2.248	2.251	2.221
Theoretical g_z_ factor	2.372	2.387	2.376	2.386
Experimental g factor	2.275	2.249	2.304	2.262
Theoretical g factor (%)	97.4	98.9	96.3	98.1

**Table 15:** Comparison between the results of calculations using 40 doublet roots and those using 5 doublets for axial zero-field splitting (ZFS) D and transverse ZFS E from the second order perturbation calculation of spin–orbit coupling (SOC) and Effective Hamiltonian with CASSCF/Def2SVP method using ORCA package (version 5.01). D-tensor spin–orbit coupling is calculated using Pederson–Khanna (PK) algorithm (Unit: cm^−1^).

Complex	1	2	3	4	Mean
Number of Atoms	57	69	78	84	
2nd Order Perturbation D 40 Roots	32.118	34.468	35.724	31.106	
2nd Order Perturbation D 5 Roots	28.424	30.995	30.175	27.561	
2nd Order Perturbation D 5 Roots %	88.5%	89.9%	84.5%	88.6%	87.88%
Effective Hamiltonian D 40 Roots	29.233	31.220	32.392	28.173	
Effective Hamiltonian D 5 Roots	25.896	28.196	27.249	25.040	
Effective Hamiltonian D 5 Roots %	88.6%	90.3%	84.1%	88.9%	87.89%
2nd Order Perturbation E 40 Roots	6.837	6.687	5.980	8.125	
2nd Order Perturbation E 5 Roots	6.508	6.689	4.853	8.092	
2nd Order Perturbation E 5 Roots %	95.2%	100%	81.2%	99.6%	94.00%
Effective Hamiltonian E 40 Roots	6.472	6.300	5.689	7.637	
Effective Hamiltonian E 5 Roots	5.989	6.203	4.212	7.524	
Effective Hamiltonian E 5 Roots %	92.5%	98.5%	74.0%	98.5%	97.88%

## Data Availability

Data are contained within the article.

## References

[1] Coronado E. (2020). Molecular magnetism: From chemical design to spin control in molecules, materials and devices. Nat. Rev. Mater..

[2] Zhu Z., Tang J. (2022). Lanthanide single-molecule magnets with high anisotropy barrier: Where to from here?. Natl. Sci. Rev..

[3] Zhu Z., Guo M., Li X.-L., Tang J. (2019). Molecular magnetism of lanthanide: Advances and perspectives. Coord. Chem. Rev..

[4] Mautner F.A., Bierbaumer F., Fischer R.C., Tubau À., Speed S., Ruiz E., Massoud S.S., Vicente R., Gómez-Coca S. (2022). Insights into the Spin Dynamics of Mononuclear Cerium(III) Single-Molecule Magnets. Inorg. Chem..

[5] Shao D., Wang X.-Y. (2020). Development of Single-Molecule Magnets†. Chin. J. Chem..

[6] Feng M., Tong M.-L. (2018). Single Ion Magnets from 3d to 5f: Developments and Strategies. Chem. A Eur. J..

[7] Craig G.A., Murrie M. (2015). 3d single-ion magnets. Chem. Soc. Rev..

[8] Zadrozny J.M., Xiao D.J., Atanasov M., Long G.J., Grandjean F., Neese F., Long J.R. (2013). Magnetic blocking in a linear iron(I) complex. Nat. Chem..

[9] Kumar Sahu P., Kharel R., Shome S., Goswami S., Konar S. (2023). Understanding the unceasing evolution of Co(II) based single-ion magnets. Coord. Chem. Rev..

[10] Guo F.S., Day B.M., Chen Y.C., Tong M.L., Mansikkamäki A., Layfield R.A. (2017). A Dysprosium Metallocene Single-Molecule Magnet Functioning at the Axial Limit. Angew. Chem. Int. Ed. Engl..

[11] Yin X., Deng L., Ruan L., Wu Y., Luo F., Qin G., Han X., Zhang X. (2023). Recent Progress for Single-Molecule Magnets Based on Rare Earth Elements. Materials.

[12] Sessoli R., Gatteschi D., Caneschi A., Novak M.A. (1993). Magnetic bistability in a metal-ion cluster. Nature.

[13] Wernsdorfer W., Sellmyer D., Skomski R. (2006). Molecular Nanomagnets. Advanced Magnetic Nanostructures.

[14] Freedman D.E., Harman W.H., Harris T.D., Long G.J., Chang C.J., Long J.R. (2010). Slow Magnetic Relaxation in a High-Spin Iron(II) Complex. J. Am. Chem. Soc..

[15] Mannini M., Pineider F., Sainctavit P., Danieli C., Otero E., Sciancalepore C., Talarico A.M., Arrio M.A., Cornia A., Gatteschi D. (2009). Magnetic memory of a single-molecule quantum magnet wired to a gold surface. Nat. Mater..

[16] Moreno-Pineda E., Wernsdorfer W. (2021). Measuring molecular magnets for quantum technologies. Nat. Rev. Phys..

[17] Greenspon A.S., Marceaux B.L., Hu E.L. (2018). Robust lanthanide emitters in polyelectrolyte thin films for photonic applications. Nanotechnology.

[18] Boulon M.-E., Cucinotta G., Luzon J., Degl’Innocenti C., Perfetti M., Bernot K., Calvez G., Caneschi A., Sessoli R. (2013). Magnetic Anisotropy and Spin-Parity Effect Along the Series of Lanthanide Complexes with DOTA. Angew. Chem. Int. Ed..

[19] Woodruff D.N., Winpenny R.E.P., Layfield R.A. (2013). Lanthanide Single-Molecule Magnets. Chem. Rev..

[20] Yatoo M., Habib F., Ahmad Z., Ahmad S., Husain A. (2023). A Concise Review of Single-Molecule Magnets, Carbon Nanotubes and Hybrids Between Them. Preprints.

[21] Duan Y., Rosaleny L.E., Coutinho J.T., Giménez-Santamarina S., Scheie A., Baldoví J.J., Cardona-Serra S., Gaita-Ariño A. (2022). Data-driven design of molecular nanomagnets. Nat. Commun..

[22] Etesse J., Holzäpfel A., Ortu A., Afzelius M. (2021). Optical and spin manipulation of non-Kramers rare-earth ions in a weak magnetic field for quantum memory applications. Phys. Rev. A.

[23] Mondal S., Lunghi A. (2022). Unraveling the Contributions to Spin-Lattice Relaxation in Kramers Single-Molecule Magnets. J. Am. Chem. Soc..

[24] Pedersen K.S., Sigrist M., Sørensen M.A., Barra A.-L., Weyhermüller T., Piligkos S., Thuesen C.A., Vinum M.G., Mutka H., Weihe H. (2014). [ReF6]2−: A Robust Module for the Design of Molecule-Based Magnetic Materials. Angew. Chem. Int. Ed..

[25] Sato R., Suzuki K., Minato T., Shinoe M., Yamaguchi K., Mizuno N. (2015). Field-induced slow magnetic relaxation of octahedrally coordinated mononuclear Fe(iii)-, Co(ii)-, and Mn(iii)-containing polyoxometalates. Chem. Commun..

[26] Vallejo J., Castro I., Ruiz-García R., Cano J., Julve M., Lloret F., De Munno G., Wernsdorfer W., Pardo E. (2012). Field-Induced Slow Magnetic Relaxation in a Six-Coordinate Mononuclear Cobalt(II) Complex with a Positive Anisotropy. J. Am. Chem. Soc..

[27] Palii A.V., Korchagin D.V., Yureva E.A., Akimov A.V., Misochko E.Y., Shilov G.V., Talantsev A.D., Morgunov R.B., Aldoshin S.M., Tsukerblat B.S. (2016). Single-Ion Magnet Et4N[CoII(hfac)3] with Nonuniaxial Anisotropy: Synthesis, Experimental Characterization, and Theoretical Modeling. Inorg. Chem..

[28] Wang Y.-L., Chen L., Liu C.-M., Zhang Y.-Q., Yin S.-G., Liu Q.-Y. (2015). Field-Induced Slow Magnetic Relaxation and Gas Adsorption Properties of a Bifunctional Cobalt(II) Compound. Inorg. Chem..

[29] Meng Y.-S., Mo Z., Wang B.-W., Zhang Y.-Q., Deng L., Gao S. (2015). Observation of the single-ion magnet behavior of d8 ions on two-coordinate Co(i)–NHC complexes. Chem. Sci..

[30] Liu X., Sun L., Zhou H., Cen P., Jin X., Xie G., Chen S., Hu Q. (2015). Single-Ion-Magnet Behavior in a Two-Dimensional Coordination Polymer Constructed from CoII Nodes and a Pyridylhydrazone Derivative. Inorg. Chem..

[31] Mondal A.K., Jover J., Ruiz E., Konar S. (2017). Investigation of easy-plane magnetic anisotropy in P-ligand square-pyramidal CoII single ion magnets. Chem. Commun..

[32] Mondal A.K., Goswami T., Misra A., Konar S. (2017). Probing the Effects of Ligand Field and Coordination Geometry on Magnetic Anisotropy of Pentacoordinate Cobalt(II) Single-Ion Magnets. Inorg. Chem..

[33] Cahier B., Perfetti M., Zakhia G., Naoufal D., El-Khatib F., Guillot R., Rivière E., Sessoli R., Barra A.-L., Guihéry N. (2017). Magnetic Anisotropy in Pentacoordinate NiII and CoII Complexes: Unraveling Electronic and Geometrical Contributions. Chem. A Eur. J..

[34] El-Khatib F., Cahier B., Shao F., López-Jordà M., Guillot R., Rivière E., Hafez H., Saad Z., Girerd J.-J., Guihéry N. (2017). Design and Magnetic Properties of a Mononuclear Co(II) Single Molecule Magnet and Its Antiferromagnetically Coupled Binuclear Derivative. Inorg. Chem..

[35] Shao D., Shi L., Zhang S.-L., Zhao X.-H., Wu D.-Q., Wei X.-Q., Wang X.-Y. (2016). Syntheses, structures, and magnetic properties of three new chain compounds based on a pentagonal bipyramidal Co(ii) building block. CrystEngComm.

[36] Świtlicka-Olszewska A., Palion-Gazda J., Klemens T., Machura B., Vallejo J., Cano J., Lloret F., Julve M. (2016). Single-ion magnet behaviour in mononuclear and two-dimensional dicyanamide-containing cobalt(ii) complexes. Dalton Trans..

[37] Briganti M., Santanni F., Tesi L., Totti F., Sessoli R., Lunghi A. (2021). A Complete Ab Initio View of Orbach and Raman Spin–Lattice Relaxation in a Dysprosium Coordination Compound. J. Am. Chem. Soc..

[38] Accorsi S., Barra A.-L., Caneschi A., Chastanet G., Cornia A., Fabretti A.C., Gatteschi D., Mortalò C., Olivieri E., Parenti F. (2006). Tuning Anisotropy Barriers in a Family of Tetrairon(III) Single-Molecule Magnets with an S = 5 Ground State. J. Am. Chem. Soc..

[39] Lu F., Guo W.-X., Zhang Y.-Q. (2022). Largely Enhancing the Blocking Energy Barrier and Temperature of a Linear Cobalt(II) Complex through the Structural Distortion: A Theoretical Exploration. Inorg. Chem..

[40] Murrie M. (2010). Cobalt(ii) single-molecule magnets. Chem. Soc. Rev..

[41] Oshio H., Nakano M. (2005). High-spin molecules with magnetic anisotropy toward single-molecule magnets. Chemistry.

[42] Fukui K., Ohya-Nishiguchi H., Hirota N. (1990). The relation between zero-field splittings and distortions along the normal coordinates in transition metal complexes. Mol. Phys..

[43] Murugesan S., Stöger B., Carvalho M.D., Ferreira L.P., Pittenauer E., Allmaier G., Veiros L.F., Kirchner K. (2014). Synthesis and Reactivity of Four- and Five-Coordinate Low-Spin Cobalt(II) PCP Pincer Complexes and Some Nickel(II) Analogues. Organometallics.

[44] Krzystek J., Zvyagin S.A., Ozarowski A., Fiedler A.T., Brunold T.C., Telser J. (2004). Definitive Spectroscopic Determination of Zero-Field Splitting in High-Spin Cobalt(II). J. Am. Chem. Soc..

[45] Devkota L., SantaLucia D.J., Wheaton A.M., Pienkos A.J., Lindeman S.V., Krzystek J., Ozerov M., Berry J.F., Telser J., Fiedler A.T. (2023). Spectroscopic and Magnetic Studies of Co(II) Scorpionate Complexes: Is There a Halide Effect on Magnetic Anisotropy?. Inorg. Chem..

[46] Kremer S., Henke W., Reinen D. (1982). High-spin-low-spin equilibriums of cobalt(2+) in the terpyridine complexes Co(terpy)2X2.nH2O. Inorg. Chem..

[47] Izumiyama N., Fujii S., Kato K., Tokunaga R., Hayami S., Nakaya M. (2024). Spin-crossover cobalt(ii) complexes exhibiting temperature- and concentration-dependent optical changes in solution. Dalton Trans..

[48] Craven M., Nygaard M.H., Zadrozny J.M., Long J.R., Overgaard J. (2018). Determination of d-Orbital Populations in a Cobalt(II) Single-Molecule Magnet Using Single-Crystal X-ray Diffraction. Inorg. Chem..

[49] Palii A.V., Clemente-Juan J.M., Coronado E., Klokishner S.I., Ostrovsky S.M., Reu O.S. (2010). Role of Orbital Degeneracy in the Single Molecule Magnet Behavior of a Mononuclear High-Spin Fe(II) Complex. Inorg. Chem..

[50] Gomez-Coca S., Cremades E., Aliaga-Alcalde N., Ruiz E. (2013). Mononuclear single-molecule magnets: Tailoring the magnetic anisotropy of first-row transition-metal complexes. J. Am. Chem. Soc..

[51] Bunting P.C., Atanasov M., Damgaard-Møller E., Perfetti M., Crassee I., Orlita M., Overgaard J., van Slageren J., Neese F., Long J.R. (2018). A linear cobalt(II) complex with maximal orbital angular momentum from a non-Aufbau ground state. Science.

[52] Yao X.N., Du J.Z., Zhang Y.Q., Leng X.B., Yang M.W., Jiang S.D., Wang Z.X., Ouyang Z.W., Deng L., Wang B.W. (2017). Two-Coordinate Co(II) Imido Complexes as Outstanding Single-Molecule Magnets. J. Am. Chem. Soc..

[53] Eichhöfer A., Lan Y., Mereacre V., Bodenstein T., Weigend F. (2014). Slow magnetic relaxation in trigonal-planar mononuclear Fe(II) and Co(II) bis(trimethylsilyl)amido complexes--a comparative study. Inorg. Chem..

[54] Rechkemmer Y., Breitgoff F.D., van der Meer M., Atanasov M., Hakl M., Orlita M., Neugebauer P., Neese F., Sarkar B., van Slageren J. (2016). A four-coordinate cobalt(II) single-ion magnet with coercivity and a very high energy barrier. Nat. Commun..

[55] Fataftah M.S., Zadrozny J.M., Rogers D.M., Freedman D.E. (2014). A Mononuclear Transition Metal Single-Molecule Magnet in a Nuclear Spin-Free Ligand Environment. Inorg. Chem..

[56] Wu C.-M., Tsai J.-E., Lee G.-H., Yang E.-C. (2020). Slow magnetization relaxation in a tetrahedrally coordinated mononuclear Co(ii) complex exclusively ligated with phenanthroline ligands. Dalton Trans..

[57] Cui H.H., Lu F., Chen X.T., Zhang Y.Q., Tong W., Xue Z.L. (2019). Zero-Field Slow Magnetic Relaxation and Hysteresis Loop in Four-Coordinate Co(II) Single-Ion Magnets with Strong Easy-Axis Anisotropy. Inorg. Chem..

[58] Ziegenbalg S., Hornig D., Görls H., Plass W. (2016). Cobalt(II)-Based Single-Ion Magnets with Distorted Pseudotetrahedral [N2O2] Coordination: Experimental and Theoretical Investigations. Inorg. Chem..

[59] Ferentinos E., Tzeli D., Sottini S., Groenen E., Ozerov M., Poneti G., Kaniewska-Laskowska K., Krzystek J., Kyritsis P. (2023). Magnetic anisotropy and structural flexibility in the field-induced single ion magnets [Co{(OPPh 2 )(EPPh 2 )N} 2 ], E = S, Se, explored by experimental and computational methods. Dalton Trans..

[60] Massoud S.S., Perez Z.E., Courson J.R., Fischer R.C., Mautner F.A., Vančo J., Čajan M., Trávníček Z. (2020). Slow magnetic relaxation in penta-coordinate cobalt(ii) field-induced single-ion magnets (SIMs) with easy-axis magnetic anisotropy. Dalton Trans..

[61] Mora-Fonz M.J., Catlow C.R., Lewis D.W. (2005). Oligomerization and cyclization processes in the nucleation of microporous silicas. Angew. Chem. Int. Ed. Engl..

[62] Jurca T., Farghal A., Lin P.H., Korobkov I., Murugesu M., Richeson D.S. (2011). Single-molecule magnet behavior with a single metal center enhanced through peripheral ligand modifications. J. Am. Chem. Soc..

[63] Bamberger H., Albold U., Dubnická Midlíková J., Su C.-Y., Deibel N., Hunger D., Hallmen P.P., Neugebauer P., Beerhues J., Demeshko S. (2021). Iron(II), Cobalt(II), and Nickel(II) Complexes of Bis(sulfonamido)benzenes: Redox Properties, Large Zero-Field Splittings, and Single-Ion Magnets. Inorg. Chem..

[64] Mitsuhashi R., Hosoya S., Suzuki T., Sunatsuki Y., Sakiyama H., Mikuriya M. (2019). Hydrogen-bonding interactions and magnetic relaxation dynamics in tetracoordinated cobalt(ii) single-ion magnets. Dalton Trans..

[65] Böhme M., Ziegenbalg S., Aliabadi A., Schnegg A., Görls H., Plass W. (2018). Magnetic relaxation in cobalt(ii)-based single-ion magnets influenced by distortion of the pseudotetrahedral [N2O2] coordination environment. Dalton Trans..

[66] Plyuta N., Petrusenko S., Kokozay V.N., Cauchy T., Lloret F., Julve M., Cano J., Avarvari N. (2022). Field-induced mononuclear cobalt(II) single-molecule magnet (SMM) based on a benzothiadiazole-ortho-vanillin ligand †. Dalton Trans..

[67] Landart-Gereka A., Quesada-Moreno M.M., Díaz-Ortega I.F., Nojiri H., Ozerov M., Krzystek J., Palacios M.A., Colacio E. (2022). Large easy-axis magnetic anisotropy in a series of trigonal prismatic mononuclear cobalt(ii) complexes with zero-field hidden single-molecule magnet behaviour: The important role of the distortion of the coordination sphere and intermolecular interactions in the slow relaxation. Inorg. Chem. Front..

[68] Tupolova Y.P., Shcherbakov I.N., Korchagin D.V., Tkachev V.V., Lebedev V.E., Popov L.D., Zakharov K.V., Vasiliev A.N., Palii A.V., Aldoshin S.M. (2020). Fine-Tuning of Uniaxial Anisotropy and Slow Relaxation of Magnetization in the Hexacoordinate Co(II) Complexes with Acidoligands. J. Phys. Chem. C.

[69] Mičová R., Rajnák C., Titiš J., Bieńko A., Moncoľ J., Samoľová E., Boča R. (2023). Easy-axis magnetic anisotropy in tetragonally elongated cobalt(ii) complexes beyond the spin-Hamiltonian formalism. Dalton Trans..

[70] Pilichos E., Font-Bardia M., Cano J., Escuer A., Mayans J. (2022). Slow magnetic relaxation for cobalt(ii) complexes in axial bipyramidal environment: An S = 1/2 spin case. Dalton Trans..

[71] Mondal A.K., Mondal A., Konar S. (2020). Slow Magnetic Relaxation in a One-Dimensional Coordination Polymer Constructed from Hepta-Coordinate Cobalt(II) Nodes. Magnetochemistry.

[72] Shao D., Zhang S.-L., Shi L., Zhang Y.-Q., Wang X.-Y. (2016). Probing the Effect of Axial Ligands on Easy-Plane Anisotropy of Pentagonal-Bipyramidal Cobalt(II) Single-Ion Magnets. Inorg. Chem..

[73] Chen L., Wang J., Wei J.-M., Wernsdorfer W., Chen X.-T., Zhang Y.-Q., Song Y., Xue Z.-L. (2014). Slow magnetic relaxation in a mononuclear eight-coordinate cobalt(II) complex. J. Am. Chem. Soc..

[74] Ruamps R., Batchelor L.J., Maurice R., Gogoi N., Jiménez-Lozano P., Guihéry N., de Graaf C., Barra A.-L., Sutter J.-P., Mallah T. (2013). Origin of the Magnetic Anisotropy in Heptacoordinate NiII and CoII Complexes. Chem. A Eur. J..

[75] Kopotkov V.A., Korchagin D.V., Sasnovskaya V.D., Gilmutdinov I.F., Yagubskii E.B. (2019). A Series of Field-Induced Single-Ion Magnets Based on the Seven-Coordinate Co(II) Complexes with the Pentadentate (N_3_O_2_) H2dapsc Ligand. Magnetochemistry.

[76] Habib F., Luca O.R., Vieru V., Shiddiq M., Korobkov I., Gorelsky S.I., Takase M.K., Chibotaru L.F., Hill S., Crabtree R.H. (2013). Influence of the Ligand Field on Slow Magnetization Relaxation versus Spin Crossover in Mononuclear Cobalt Complexes. Angew. Chem. Int. Ed..

[77] Massoud S.S., Mautner F.A., Sakiyama H., Louka F.R., Salem N.H.M., Fischer R.C., Torvisco A., Guizouarn T., Velmurugan G., Comba P. (2025). SMM Behavior in Distorted Trigonal Bipyramidal and Tetrahedral Cobalt(II) Complexes Based on Tripodal Tetradentate Phenolic Amines. Eur. J. Inorg. Chem..

[78] Dong X., Xin Z., He D., Zhang J.-L., Lan Y.-Q., Zhang Q.-F., Chen Y. (2023). Boosting CO2 electroreduction performance over fullerene-modified MOF-545-Co promoted by π–π interaction. Chin. Chem. Lett..

[79] Subedi D., Jang Y., Ganesan A., Schoellhorn S., Reid R., Verbeck G., D’Souza F. (2021). Donor-acceptor conjugates derived from cobalt porphyrin and fullerene via metal-ligand axial coordination: Formation and excited state charge separation. J. Porphyr. Phthalocyanines.

[80] Liu Y., Gallo A.A., Liu Y., Hall M.B., Johnson B.R. (2023). QM evaluation of the intramolecular aromatic π-π interactions of Ir(I) complex transition states. J. Mol. Struct..

[81] Lunghi A., Sanvito S. (2022). Computational design of magnetic molecules and their environment using quantum chemistry, machine learning and multiscale simulations. Nat. Rev. Chem..

[82] Frisch M.J., Trucks G.W., Schlegel H.B., Scuseria G.E., Robb M.A., Cheeseman J.R., Scalmani G., Barone V., Petersson G.A., Nakatsuji H. (2016). Gaussian 16 Rev. C.01.

[83] Neese F. (2012). The ORCA program system. WIREs Comput. Mol. Sci..

[84] Perdew J.P. (1986). Density-functional approximation for the correlation energy of the inhomogeneous electron gas. Phys. Rev. B.

[85] Becke A.D. (1986). Density functional calculations of molecular bond energies. J. Chem. Phys..

[86] Becke A.D., Johnson E.R. (2005). A density-functional model of the dispersion interaction. J. Chem. Phys..

[87] Weigend F., Ahlrichs R. (2005). Balanced basis sets of split valence, triple zeta valence and quadruple zeta valence quality for H to Rn: Design and assessment of accuracy. Phys. Chem. Chem. Phys..

[88] Neese F., Wennmohs F., Hansen A., Becker U. (2009). Efficient, approximate and parallel Hartree–Fock and hybrid DFT calculations. A ‘chain-of-spheres’ algorithm for the Hartree–Fock exchange. Chem. Phys..

[89] Weigend F. (2006). Accurate Coulomb-fitting basis sets for H to Rn. Phys. Chem. Chem. Phys..

[90] Jung J., Atanasov M., Neese F. (2017). Ab Initio Ligand-Field Theory Analysis and Covalency Trends in Actinide and Lanthanide Free Ions and Octahedral Complexes. Inorg. Chem..

[91] Angeli C., Cimiraglia R., Evangelisti S., Leininger T., Malrieu J.P. (2001). Introduction of n-electron valence states for multireference perturbation theory. J. Chem. Phys..

[92] Chilton N.F., Anderson R.P., Turner L.D., Soncini A., Murray K.S. (2013). PHI: A powerful new program for the analysis of anisotropic monomeric and exchange-coupled polynuclear d- and f-block complexes. J. Comput. Chem..

[93] Glaesemann K.R., Schmidt M.W. (2010). On the Ordering of Orbital Energies in High-Spin ROHF. J. Phys. Chem. A.

[94] Zanders D., Boysen N., Land M.A., Obenlüneschloß J., Masuda J.D., Mallick B., Barry S.T., Devi A. (2021). Co(II) Amide, Pyrrolate, and Aminopyridinate Complexes: Assessment of their Manifold Structural Chemistry and Thermal Properties. Eur. J. Inorg. Chem..

[95] Kim H.-C., Mitra S., Veerana M., Lim J.-S., Jeong H.-R., Park G., Huh S., Kim S.-J., Kim Y. (2019). Cobalt(II)-coordination polymers containing glutarates and bipyridyl ligands and their antifungal potential. Sci. Rep..

[96] Chin Y.-Y., Lin H.-J., Hu Z., Kuo C.-Y., Mikhailova D., Lee J.-M., Haw S.-C., Chen S.-A., Schnelle W., Ishii H. (2017). Relation between the Co-O bond lengths and the spin state of Co in layered Cobaltates: A high-pressure study. Sci. Rep..

[97] Buron-Le Cointe M., Hébert J., Baldé C., Moisan N., Toupet L., Guionneau P., Létard J.F., Freysz E., Cailleau H., Collet E. (2012). Intermolecular control of thermoswitching and photoswitching phenomena in two spin-crossover polymorphs. Phys. Rev. B.

[98] Addison A.W., Rao T.N., Reedijk J., van Rijn J., Verschoor G.C. (1984). Synthesis, structure, and spectroscopic properties of copper(II) compounds containing nitrogen–sulphur donor ligands; the crystal and molecular structure of aqua[1,7-bis(N-methylbenzimidazol-2′-yl)-2,6-dithiaheptane]copper(II) perchlorate. J. Chem. Soc. Dalton Trans..

[99] Yang L., Powell D.R., Houser R.P. (2007). Structural variation in copper(i) complexes with pyridylmethylamide ligands: Structural analysis with a new four-coordinate geometry index, τ4. Dalton Trans..

[100] Alonso J.A., Martínez-Lope M.J., Casais M.T., Fernández-Díaz M.T. (2000). Evolution of the Jahn−Teller Distortion of MnO6 Octahedra in RMnO3 Perovskites (R = Pr, Nd, Dy, Tb, Ho, Er, Y): A Neutron Diffraction Study. Inorg. Chem..

[101] Marchivie M., Guionneau P., Letard J.-F., Chasseau D. (2005). Photo-induced spin-transition: The role of the iron(II) environment distortion. Acta Crystallogr. Sect. B.

[102] McCusker J.K., Rheingold A.L., Hendrickson D.N. (1996). Variable-Temperature Studies of Laser-Initiated 5T2 → 1A1 Intersystem Crossing in Spin-Crossover Complexes:  Empirical Correlations between Activation Parameters and Ligand Structure in a Series of Polypyridyl Ferrous Complexes. Inorg. Chem..

[103] Köppel H., Barentzen H. (2009). The Jahn-Teller Effect: Fundamentals and Implications for Physics and Chemistry.

[104] Okuniewski A., Rosiak D., Chojnacki J., Becker B. (2015). Coordination polymers and molecular structures among complexes of mercury(II) halides with selected 1-benzoylthioureas. Polyhedron.

[105] Šebová M., Boca R., Dlhan L., Nemec I., Papankova B., Pavlik J., Fuess H. (2012). Direct determination of zero-field splitting in Co(II) complexes by FAR infrared spectroscopy. Inorganica Chim. Acta.

[106] Weinhold F., Landis C.R., Glendening E.D. (2016). What is NBO analysis and how is it useful?. Int. Rev. Phys. Chem..

[107] Weinhold F. (2012). Natural bond orbital analysis: A critical overview of relationships to alternative bonding perspectives. J. Comput. Chem..

[108] Mizukami S., Houjou H., Sugaya K., Koyama E., Tokuhisa H., Sasaki T., Kanesato M. (2005). Fluorescence Color Modulation by Intramolecular and Intermolecular π−π Interactions in a Helical Zinc(II) Complex. Chem. Mater..

[109] Liu Y., Liu Y., Murru S., Tzeng N., Srivastava R.S. (2015). Quantum mechanics study of repulsive π–π interaction and flexibility of phenyl moiety in the iron azodioxide complex. J. Mol. Struct..

[110] Ciliberto E., Doris K.A., Pietro W.J., Reisner G.M., Ellis D.E., Fragala I., Herbstein F.H., Ratner M.A., Marks T.J. (1984). The.pi.-.pi. interactions and bandwidths in molecular metals. A chemical, structural, photoelectron spectroscopic, and Hartree-Fock-Slater study of monomeric and cofacially joined dimeric silicon phthalocyanines. J. Am. Chem. Soc..

[111] Carter-Fenk K., Liu M., Pujal L., Loipersberger M., Tsanai M., Vernon R.M., Forman-Kay J.D., Head-Gordon M., Heidar-Zadeh F., Head-Gordon T. (2023). The Energetic Origins of Pi-Pi Contacts in Proteins. J. Am. Chem. Soc..

[112] Kowalski P.H., Krzemińska A., Pernal K., Pastorczak E. (2022). Dispersion Interactions between Molecules in and out of Equilibrium Geometry: Visualization and Analysis. J. Phys. Chem. A.

[113] Khare E., Holten-Andersen N., Buehler M.J. (2021). Transition-metal coordinate bonds for bioinspired macromolecules with tunable mechanical properties. Nat. Rev. Mater..

[114] Laporte O., Meggers W.F. (1925). Some Rules of Spectral Structure*. J. Opt. Soc. Am..

[115] Schnaubelt L., Petzold H., Speck J.M., Rüffer T., Hörner G., Lang H. (2018). Spin Transition and Charge Transfer in Co2+/Co3+ Complexes of Meridional Ligands Holding Nearby Redox-active Triarylamine. Z. Anorg. Allg. Chem..

[116] May A.M., Dempsey J.L. (2024). A new era of LMCT: Leveraging ligand-to-metal charge transfer excited states for photochemical reactions. Chem. Sci..

[117] Tuszyński J.A. (1986). First- and second-order contributions to the effective Hamiltonian parameters for Gd3+(8S) and Mn2+(6S). J. Magn. Magn. Mater..

[118] Kumar S., Arumugam S., Schwarz B., Ehrenberg H., Mondal K. (2023). Static and Dynamic Magnetic Properties of a Co(II)-Complex with N_2_O_2_ Donor Set—A Theoretical and Experimental Study. Eur. J. Inorg. Chem..

[119] Rao S.V., Maganas D., Sivalingam K., Atanasov M., Neese F. (2024). Extended Active Space Ab Initio Ligand Field Theory: Applications to Transition-Metal Ions. Inorg. Chem..

[120] Lloret F., Julve M., Cano J., Ruiz-García R., Pardo E. (2008). Magnetic properties of six-coordinated high-spin cobalt(II) complexes: Theoretical background and its application. Inorganica Chim. Acta.

[121] Khosla A.L., Jacko A.C., Merino J., Powell B.J. (2017). Spin-orbit coupling and strong electronic correlations in cyclic molecules. Phys. Rev. B.

[122] Šulka M., Šulková K., Dubecký M. (2024). Unveiling hidden dynamic correlations in CASSCF correlation energies by Hartree–Fock nodes. J. Chem. Phys..

[123] Landé A. (1921). Über den anomalen Zeemaneffekt (Teil I). Z. Phys..

[124] Sakiyama H., Kimura R., Oomiya H., Mitsuhashi R., Fujii S., Kanaizuka K., Muddassir M., Tamaki Y., Asato E., Handa M. (2024). Relationship between Structure and Zero-Field Splitting of Octahedral Nickel(II) Complexes with a Low-Symmetric Tetradentate Ligand. Magnetochemistry.

[125] Pankratova Y.A., Nelyubina Y.V., Novikov V.V., Pavlov A.A. (2021). High-Spin Cobalt(II) Complex with Record-Breaking Anisotropy of the Magnetic Susceptibility According to Paramagnetic NMR Spectroscopy Data. Russ. J. Coord. Chem..

